# Two way workable microchanneled hydrogel suture to diagnose, treat and monitor the infarcted heart

**DOI:** 10.1038/s41467-024-45144-y

**Published:** 2024-01-29

**Authors:** Fangchao Xue, Shanlan Zhao, Hao Tian, Haoxiang Qin, Xiaochen Li, Zhao Jian, Jiahui Du, Yanzhao Li, Yanhong Wang, Lin Lin, Chen Liu, Yongning Shang, Lang He, Malcolm Xing, Wen Zeng

**Affiliations:** 1 Department of Cell Biology, Third Military Medical University, Chongqing, China; 2 Department of Cardiovascular Surgery, Xinqiao Hospital, Third Military Medical University, Chongqing, China; 3 Department of Anatomy, Third Military Medical University, Chongqing, China; 4 Department of Radiology, Southwest hospital, Third Military Medical University, Chongqing, China; 5 Department of Ultrasound, Southwest hospital, Third Military Medical University, Chongqing, China; 6 Department of Mechanical Engineering University of Manitoba, Winnipeg, Canada; 7 State Key Laboratory of Trauma and Chemical Poisoning, Chongqing, China; 8 Jinfeng Laboratory, Chongqing, People’s Republic of China

**Keywords:** Tissue engineering, Biomaterials, Biotechnology, Biomaterials, Materials for devices

## Abstract

During myocardial infarction, microcirculation disturbance in the ischemic area can cause necrosis and formation of fibrotic tissue, potentially leading to malignant arrhythmia and myocardial remodeling. Here, we report a microchanneled hydrogel suture for two-way signal communication, pumping drugs on demand, and cardiac repair. After myocardial infarction, our hydrogel suture monitors abnormal electrocardiogram through the mobile device and triggers nitric oxide on demand via the hydrogel sutures’ microchannels, thereby inhibiting inflammation, promoting microvascular remodeling, and improving the left ventricular ejection fraction in rats and minipigs by more than 60% and 50%, respectively. This work proposes a suture for bidirectional communication that acts as a cardio-patch to repair myocardial infarction, that remotely monitors the heart, and can deliver drugs on demand.

## Abstract (web summary)

Myocardial infarction can lead to malignant arrhythmia and myocardial remodeling. Here, the authors report a microchanneled hydrogel suture for two-way signal communication, pumping drugs on demand, and cardiac repair.

## Introduction

Our lives hinge on organs and tissues to work accurately around the clock. They may fail when damaged, and organ transplantation is needed. However, donor organ scarcity leads to the increasing demand for repairing and regeneration^1,2^. Heart disease is a leading cause of death and contributes a substantial burden to our society. Heart attack is unexpected but comes about instantly^3^. During myocardial infarction (MI), microcirculation disturbance in the ischemic area leads to tissue necrosis and fibrous tissue forming, leading to malignant arrhythmia and myocardial remodeling, affecting the function of the myocardium and reducing the quality of life of patients^4^.

The timely detection, feedback, and corresponding treatment of malignant events are still challenging. Myocardial patches with elasticity and injectability show substantial advances in evidence-based therapy to treat MI. However, surgical complex and adhesive-aided patches during injection complicate the aggregability in practice^5–7^. On the other hand, myocardial patches can preload with drugs or cells and slowly release cells or factors according to their design. But the cargo release from the patch is usually of unrepeatability and non-selectivity and inept handling of disease^8^. The monitoring and evaluation of disease prognosis usually rely on complex and expensive clinical instruments. Sensors that monitor tissue lesions in real-time and evaluate the effect of treatment, timely feedback, and intervention in adverse events after surgery or discharge are badly needed^9–11^. Current cardiac sensor devices can record the electrical activities of the heart, and the array of electrodes was used to realize myocardial electrical stimulation and drug release^12,13^. Notwithstanding those groundbreaking works, there are some concerns to be addressed. When the patches or sensors are implanted on a slippery and wet heart tissue surface, the in-situ stability is unmet under a dynamic beating environment^14^. Besides, the complex procedures in cardio patch electronics with myriad etching processes and involved semiconductive materials matter the functions when a long-term setup in the heart is needed^15^. Therefore, an economic cell-free material treatment with flexibility by conforming to the heart tissue, which conveys an integrated diagnostic-treatment-monitoring function along with drug release, is a necessity^16^.

**Fig. 1:**
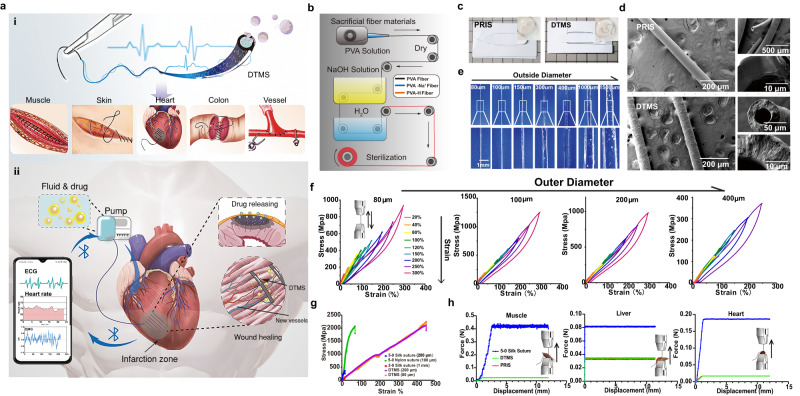
Concept design, preparation, and physical characterization of DTMS. **a** The diagnosis, treatment, and monitoring suture (DTMS) in the scheme for different tissues (i), DTMS conducting signals of infarcted hear and delivering drugs on demand (ii). **b** Manufacturing process of PVA hydrogel suture. **c** DTMS and PRIS (Primary Suture). **d** Scanning electron microscope (SEM) of DTMS and PRIS (Primary Suture), *n* = 4 independent replications with similar results. **e** Transparent PRIS with outer diameter (OD) of 80 μm, 150 μm, 300 μm, 400 μm, 1000 μm, 1.5 mm and inner diameter (ID) of 50 μm, 80 μm, 150 μm, 300 μm, 400 μm, 1000 μm. **f** Loading and unloading curve of DTMS with OD = 100,200 and 400 μm with the same ID of 80 μm. **g** Breaking strength of DTMS (ID = 50 μm, OD = 80 and 200 μm), silk suture® (diameter = 200 μm and 1 mm), nylon suture® (diameter = 100 μm) **h** Pulling resistance test of sutures from skeletal muscle, liver, and myocardium. (DTMS and PRIS with ID = 80 μm and OD = 200 μm), silk suture® (diameter = 200 μm).

In this work, inspired by the natural vasculature, based on its perfusible, tissue-wide distribution, and sense of vascular integrity, we reported a perfusible, multifunctional vascular-liked system, which is a microchanneled hydrogel suture (Fig. 1a). Recently, functional suture had enormous potential for real-time diagnosis, evaluation, and treatment of damaged organs and tissues along its wound closure task. These sutures can realize wireless sensing, drug elution, near-infrared light heat conversion, inhibition of bacterial breeding and sensing functions. However, although these sutures can provide flexible treatment schemes, their single function in monitoring the disease’s progress of deep tissues is still limited, and it is impossible to integrate multiple monitoring schemes and provide flexible treatment (Tables S1 and S2)^11,17–21^. Here, we named this multifunction hydrogel suture as diagnosis, treatment, and monitoring suture (DTMS), which can sense real-time signals of the anastomosed wound tissue after suturing and treating, monitoring on demand. DTMS is a microchanneled polyvinyl alcohol hydrogel suture with conductive polypyrrole (Ppy). Deprotonated PVA chains form a hydrogel with a low swelling rate and flexible elasticity through hydrogen bonds^22^. Due to its efficient conductivity and unique physical structure, DTMS were used both as receptors to receive motor or bioelectric signals, and as effectors for disease intervention. DTMS is connected with a micro pump for liquid input, then the sutures with microchannels can be used for tissue-programmed drug delivery. Therefore, we first verified the abundant potential of hydrogel sutures in tissue anastomosis on the skin, muscles, intestine, and blood vessels of rats. Then, in MI rats and mini pigs, DTMS continuously sensed electrophysiological changes in injured tissue on mobile phones and effectively reduced the area of necrotic tissue in myocardial infarction, saving the decline in cardiac function.

## Results

### DTMS design

Considering that a suture’s primary requirements are strength and stability and biocompatibility, biocompatible polyvinyl alcohol (PVA) was selected. We deprotonated the polyvinyl alcohol hydroxyl group by the alkaline attack of the OH^-^ ions. A subsequent complexation between the O^-^ group and the free Na^+^ can promote the stretching and alignment of the PVA strands and form a crystalline domain^23^. Once in contact with the water, the hydrogen bond replaces the complex of the PVA chain, thus creating a crystal domain. Microcrystal formation and hydrogen bonds will expel water molecules, resulting in hydrogels with a low swelling and high strength^22^ (Fig. S1). By uniformly distributing 10% polypyrrole in PVA solution, hydrogel suture is endowed with conductivity^24^, here we named the PVA-hydrogel sutures as primary sutures (PRIS), and Ppy-PVA-hydrogel sutures as diagnosis-treatment-monitoring sutures (DTMS). The microchannel of DTMS demonstrated two-way workability for the delivery of drugs to even deep tissue and for remotely exporting the signal from tissue to outside mobile device. (Fig. 1a). Hydrogel sutures can be produced in a simple process (Fig. 1b). The DTMS and PRIS exhibited the same hydrogel and suture characteristics (Fig. 1c). In Fig. 1d, SEM showed that the PVA hydrogel sutures (PRIS) had a smooth, compact appearance, and hollow-microchannel structure. DTMS showed a mild roughness due to the addition of polypyrrole. Multi-scaled hydrogel sutures can be obtained by simply adjusting the mold in the production process, with a size (outer diameter, OD), for example, from 80 μm to 1.5 mm or even larger (Fig. 1e, Supplementary Movie 1).

### Mechanical properties of the PVA sutures

The strength of the suture is the key to ensuring that the tissue wound can be firmly closed. For sutures with OD 100–400 μm with ID = 80 μm, a cyclic stretching under a spectrum of strain from 20% to 300% was conducted. For the strain of 20% and 30%, the DTMS shows no hysteresis between loading and unloading, with linear elastic performance. Once the strain to 300%, we found dissipative energy increases, but it is still not obviously (Fig. 1f). Our suture can be designed to match soft tissues like the heart (tensile modulus ≈ 0.5 MPa)^25^, as well as tough connective tissue such as skin and muscles^26^.

The hydrogel suture can reach a significantly higher breakage strength than 2-0 and 5-0 silk sutures® and slightly higher than 5-0 nylon sutures® (*σ* = 2104.5 ± 131.2 MPa, *T* = 285.8 MJ m^−3^) (Fig. 1g). and the suture overcame the baffle of easy breakage of the commercial silk suture ® (Fig. S2). Furthermore, the hydrogel nature of the suture gives the advantage of low friction^27^. The surface roughness and friction can be markedly reduced so that the hydrogel sutures can smoothly penetrate skeletal muscle, cardiac muscle, and liver tissue without inducing damage (Fig. 1h, Figs. S3, S4)^28^. Finally, the cyclic stretching test showed that the hydrogel sutures have good elasticity and fatigue resistance (Fig. S5).

### Bidirectional perfusion functions of DTMS

**Fig. 2:**
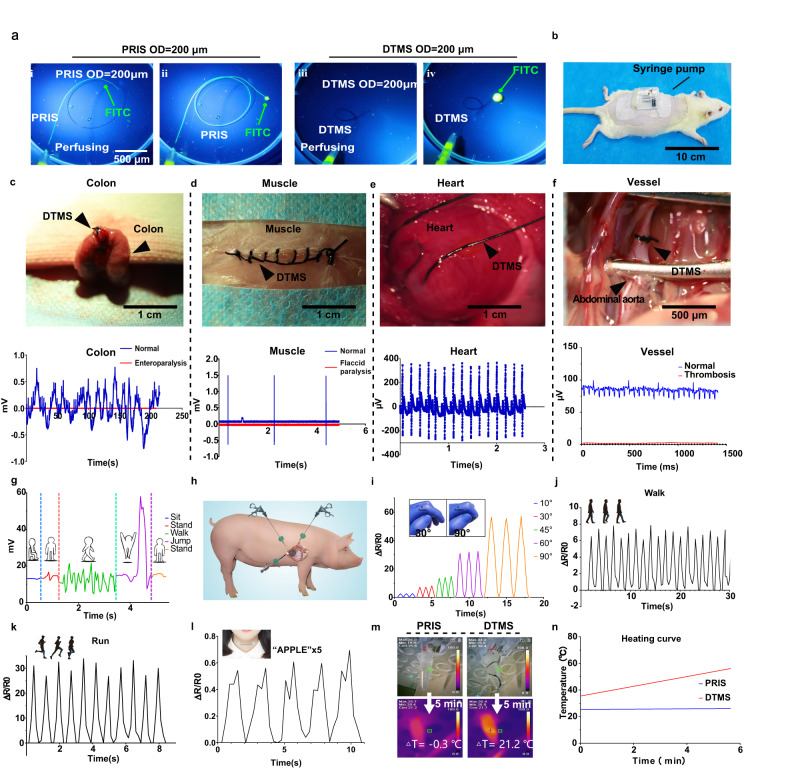
Multi-physical functions of DTMS. **a** Fluorescein 5-isothiocyanate (FITC) solution perfusion via PRIS and DTMS, OD = 200 μm, ID = 80 μm. **b** The micro pump connected DTMS on the rat. **c** DTMS and Bluetooth module sensing the electrophysiological waveform of intestinal peristalsis and detecting paralysis. **d** DTMS and Bluetooth module sensing in the skeletal muscle sensing the electromyography (EMG) of normal muscle and detecting soft paralysis. **e** DTMS and Bluetooth module in the myocardium for electrocardiogram (ECG) signal sensing. **f** DTMS in the rat abdominal aorta to monitor electrophysiological signals of vascular pulsation and detect vascular occlusion. **g** Body surface electromyography signals received by the Bluetooth module in different states. **h** Potential myocardial laparoscopic surgery using DTMS. **i**–**l** DTMS as a biomechanical sensor in joint movement figure curing **i**, walking **j**, running **k**, the vocal cords vibration **l**. **m** NIR photothermal experiment of DTMS and PRIS, **n** Heating curve of DTMS’s photothermal experiment.

The hydrogel sutures can be a fluidic channel to deliver agents via micropump, and the drug can quantitatively enter the damaged tissue in the body from the portable pump^29^ (Fig. 2a, b and Supplementary Movie 2). To deliver the agents with the potential existence of surgical knots, based on intermittent suturing, the agents were allowed to flow out of the preset orifice at one end by additional continuous suture, playing a role at the sutured site. Also, the hydrogel suture can be an extraction channel for monitoring biochemical indicators. We used commercial dynamic blood glucose meter to real-time monitor glucose levels in interstitial fluid extracted by DTMS, which was consistent with the blood glucose concentration of the peripheral blood (Fig. S6). The extraction of body fluid from the wound site provides a shortcut for early detection of infection and inflammation. Next, the drug diffusion experiment confirmed that the small molecule drugs could pass the pipeline barrier of hydrogel suture^30^ (Fig. S7). The permeability of the hydrogel makes it easier for the drug to enter the tissue.

### DTMS as a motion and bioelectric signal biosensor

Polypyrrole (PPy) gives DTMS impressive electrical conductivity (conductivity ≈ 1.47 S/cm) (Fig. S8), and DTMS can be used either for suturing or signal conducting. For normal and pathological conditions of multiple types of tissues and complex organs, DTMS can be used for tissue anastomosis and capturing tissue biological signals. The exposed conductive suture in anastomosed tissue could transmit electrical signals to the device, while the insulating PVA outer membrane and relatively fixed positions shields the DTMS from tissue and environment electromagnetic interference. For the rat skeletal muscle, the bioelectrical signals Bluetooth module recorded the EMG of the normal muscle motion and soft paralysis state (Fig. 2d). When the DTMS was applied to the colonic anastomosis, it detected the electrophysiological waveforms of bowel peristalsis and intestinal paralysis (Fig. 2c). ECG signals from the normal myocardium on the rat heart were also monitored (Fig. 2e). After anastomosis with the rat abdominal aortas, DTMS captured the electrical signal of the vascular fluctuations, which disappeared after the occlusion by thrombosis (Fig. 2f). The DTMS and bioelectrical signals Bluetooth module can remotely record the electromyography (EMG) resulting from the human motion and posture changes (Fig. 2g). As conductive elastomers, resistance-based strain sensing can reflect the state of stretch-compression motion state of hydrogel sutures. DTMS can detect the correct waveform in a single-axis tensile test (Fig. S9). In addition, DTMS is used to perceive body movements. Take example, the hydrogel suture sensed the curing of body joints, and speech recognition (Fig. 2i–l). It is worth noting that delivering drugs through another DTMS connected to the heart did not cause sensory drift, and the activities of the heart and muscles themselves did not significantly affect the monitoring of ECG signals (Fig. S10). Our suture also has the potential to integrate with the host through minimally invasive surgery (Fig. 2h).

### Force-induced luminescence effect of DTMS and photothermal conversion effect of DTMS

The ease of constructing the hydrogel sutures allowed us to enhance the function of the suture by adding additional components to the fabrication process. When constructing the hydrogel sutures, the hydrogel sutures can be endowed with force-luminescent properties by tentatively adding a ZnS:Cu of 10% mass fraction and making it evenly distributed inside the hydrogel suture (Fig. S11). The mechanical luminescence properties of ZnS:Cu have been extensively studied. Due to the bidirectional coupling effect between the piezoelectric properties and the photoexcitation properties of ZnS: Cu, the strain-induced piezoelectric potential plays an important role in bending or stretching the energy band structure within the DTMS, so as to control the process of optical excitation^31,32^. The force-luminescent hydrogel suture can emit intense fluorescence of different intensities under uniaxial stretching stimulation at different frequencies (Supplementary Movie 3). Furthermore, the photothermal conversion capacity of PPy gives the photothermal effect to DTMS. In vitro, the DTMS temperature increased to 55.4 °C after 5 min at 808 nm, and up to 45 °C after 5 min in vivo (Fig. S12, Fig. 2m, n). All results showed significant bacterial inhibition of DTMS hydrogel sutures compared to normal 5-0 and PVA hydrogel sutures for potential significant bacterial clearance of the sutures (Figs. S13, 14).

### DTMS monitoring and improving ventricular function

**Fig. 3:**
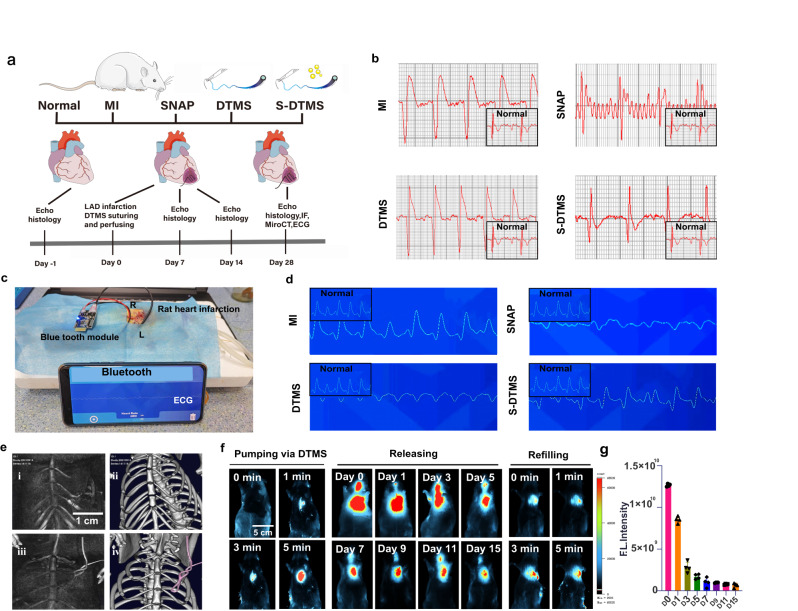
DTMS monitoring and intervening myocardial infarction (MI). **a** Scheme illustration: Normal group: normal rats; MI group: no intervention after MI; SNAP group: single SNAP treatment after MI; DTMS group: DTMS treated MI; S-DTMS group: SNAP and DTMS treated MI **b** Surface ECG signals of rats in each group measured by electrophysiology workstation after 1 month. **c, d** ECG signals of rats in each group remotely measured by DTMS and Bluetooth module after 1 month. **e** Micro-CT imaging of DTMS which passed through the chest wall and myocardial tissue, finally exited the body. (**i**) Soft tissue image prior to iohexol perfusion. (**ii**) High-density images prior to iohexol perfusion. (**iii**) Soft tissue image after iohexol perfusion. (**iv**) High-density images after iohexol perfusion. **f** Fluorescence imaging of BSA-DIR via DTMS by micro-pump on demand for two times (100 μL/min). **g** Quantification of average radiation efficiency micro-pumped DTMS perfusion. *N* = 4 biologically independent replicates. All values are presented as mean ± SD.

To evaluate the DTMS’s diagnosis-treatment-monitoring effect, we chose the acute myocardial infarction model (Fig. 3a). After assessing the biosafety and capability of DTMS (Figs. S15-16). The hydrogel suture was continuously sutured to the surface of the infarcted myocardium. And a nitric oxide (NO) donor drug, N-Acetyl-3-Nitrososulfanyl-Valine (SNAP)^33^, after determining that SNAP does not cause further oxidative damage in a reactive oxygen species environment(Fig. S17), SNAP was preloaded into a micropump on the body surface and delivered to treat MI-associated hypoxia and ischemia via our microchanneled suture. On day 30, the electrophysiology workstation was used to measure surface ECG signals of rats in each group, which is the most commonly used cardiac test in clinical practice (Fig. 3b). And the DTMS which connected to the Bluetooth module could still perceive the rat’s ECG signals. The ECG displayed on the mobile phone showed significantly S-T elevation in MI group. The trend was same as the body surface ECG collected from the electrophysiology workstation, showed the consistence of the two approaches. The sutures have the clinical potential of continuously monitoring the disease status and outcome (Fig. 3c, d, Supplementary Movie 4). Micro-CT and live fluorescence imaging presented that DTMS efficiently delivered the drug into the myocardium via microfluidic sutures. Our hydrogel suture could continuously release BSA-DIR for at least 15 days (Fig. 3e–g).

**Fig. 4:**
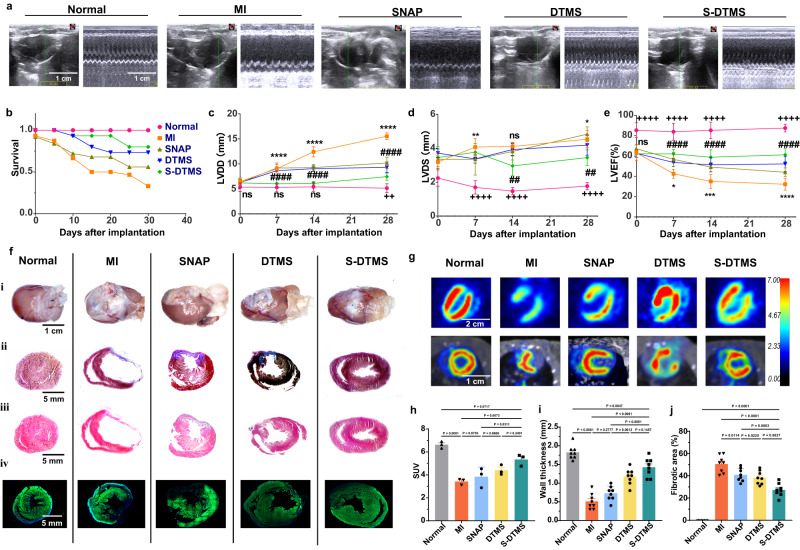
DTMS improving positively remodeling rat ventricle after MI. **a** 2D and M-mode echocardiography on day 28. **b** Survival rate of each group in 1 month. **c**–**e** Cardiac function of each group. Quantitative analysis of Left Ventricular Diastolic Dysfunction (LVDd) **c**, Left Ventricular Systolic Dimension (LVDs) **d**, and Left Heart Ejection Fraction (LVEF) **e** evaluated by echocardiography on days 0, 7, 14, 28. *N* = 5 biologically independent replicates. Two-way ANOVA with multiple comparison tests. All values are presented as mean ± SD. *####P* < 0.0001 *###P* < 0.001, *##P* < 0.01 and *#P* < 0.05, S-DTMS compared with MI group. ****P < 0.0001 and ***P < 0.001, **P < 0.01 and *P < 0.05, S-DTMS compared with DTMS group. *++++P* < 0.0001 and *+++P* < 0.001, *++P* < 0.01 and *+P* < 0.05, S-DTMS compared with Normal group. **f** (**i**) Gross images of the heart. (**ii**) Section Masson staining, (**iii**) Sliced H&E staining. (**iv**) a-SMA staining. **g** Positron emission computed tomography (PET-CT) images in cardiac long-axis and short axis on day 28. *N* = 3 biologically independent replicates. **h** Standardized uptake value (SUV) of cardiomyocytes. One-way ANOVA with multiple comparison tests. All values are presented as mean ± SD. *N* = 3 biologically independent replicates. **i**, **j** Quantitative analysis of left ventricular wall thickness **i** and myocardial fibrosis area **j** of rats after 1 month. **i**, **j** One-way ANOVA with multiple comparison tests. All values are presented as mean ± SD. *N* = 8 biologically independent replicates.

We then investigated DTMS’s therapeutic effects to improve myocardium after infarction via echocardiography (Fig. 4a). 50% higher survival in the S-DTMS group (DTMS + SNAP treatment) than in the MI group (Fig. 4b). After 1 month, the echocardiographic results showed that rats’ left ventricular function was saved in S-DTMS group. Left ventricular diastolic dysfunction (LVDd) and left ventricular systolic dimension (LVDs) was significantly prevented in S-DTMS group (Fig. 4c, d). The left heart ejection fraction (LVEF) tended to decrease slowly over time in the DTMS group (single DTMS treatment) (LVEF: 52.24 ± 7.40%) and SNAP group (single SNAP treatment) (LVEF: 44.01 ± 4.23%), and they were even lower in the MI group (LVEF:32.19 ± 5.82%). However, the S-DTMS group gradually rebounded with time (LVEF: 61.19 ± 6.30%). In the long term, compared with the MI group, the left ventricular function of rats in the S-DTMS group did not further deteriorate. The LVDD and LVDS, was significantly prevented in the S-DTMS group (LVDD: 7.59 ± 0.79 mm, LVDS: 3.22 ± 0.48 mm), compared to the MI group (LVDD: 16.8 ± 2.21 mm, LVDS: 5.49 ± 0.58 mm) at Day60 to Day90 (Fig. S18). The assessment of cardiomyocyte viability by positron emission computed tomography (PET-CT) showed that the standardized uptake values (SUV) were higher in the DTMS group (1.1 times) and S-DTMS group (1.5 times), compared to the MI group, showed more cardiomyocytes were rescued (Fig. 4g, h). We next evaluated the effect of DTMS on LV remodeling. The gross images, H&E staining, and Masson staining all showed S-DTMS group had the slightest fibrosis area (50% smaller than MI group) and thicker LV thickness (2.7 times of the MI group) (Fig. 4f, i, j). Meanwhile, the H&E staining and the smooth muscle also demonstrated that the SNAP group retained the functional cardiomyocytes (Fig. 4f). Compared with the myocardial infarction group, the group receiving our sutures reduced malignant complications such as abnormal movements in the later stages of myocardial infarction, effectively rescued dying cardiomyocytes, improved left ventricular remodeling, and improved the decline in cardiac function.

### DTMS induced reparative cell infiltration to promote tissue recovery

**Fig. 5:**
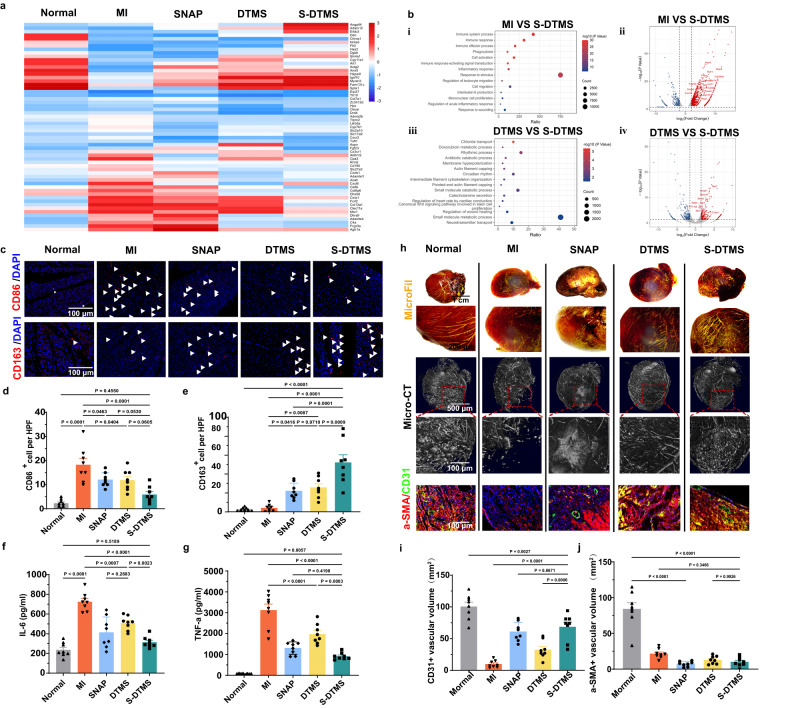
DTMS promoting angiogenesis and improving the blood supply of MI area. **a** Top differentially expressed gene heatmap of rats’ heart after 1 week with different treatments. (red, up-regulated; blue, down-regulated; *N* = 3 biologically independent replicates). **b** Transcriptome characteristics of infarcted tissue (i, iii): KEGG pathway enrichment analyze of different groups. (i) MI group vs S-DTMS group；(iii) DTMS group vs S-DTMS group. ii, iv: Top differentially expressed gene volcanic map of different groups. (ii) MI group vs S-DTMS group. (iv) DTMS group vs S-DTMS group.) *N* = 3 biologically independent replicates**. c** Macrophage immunofluorescence staining of myocardial tissue of the rat heart after 1-week treatment. The white arrows indicate the positive immunofluorescence staining macrophages. **d**, **e** Quantification of the CD86 ^+^ **d** and CD163 ^+^ **e** cells, one-way ANOVA with multiple comparison tests. All values are presented as mean ± SD. *N* = 8 biologically independent replicates. **f**, **g** Quantification of IL-6 **f**, TNF-a **g** in the myocardial tissue homogenates after 1 month. One-way ANOVA with multiple comparison tests. All values are presented as mean ± SD. *N* = 8 biologically independent replicates. **h** After 1 month, angiography and histological staining of the rat heart. From top to bottom: Overall and local features of the rat hearts’ coronary perfusion. Contrast-enhanced coronary artery micro-CT. α-SMA/CD31 immunofluorescence staining of myocardium after treatment for 1 month. **i** and **j** Quantitative analysis of the and CD31^+^ vessels **i**, α- SMA^+^ vessels **j**, one-way ANOVA with multiple comparison tests. All values are presented as mean ± SD. *N* = 8 biologically independent replicates.

To further explore our suture’s therapeutic effects in post-infarction, we performed bulk RNA sequencing on rat myocardial tissue on day 7. The genes obtained from sequencing that are differentially expressed are shown in Fig. 5a. Through further clustering and analysis of these differentially expressed genes, we found that compared with the MI group, the expression levels of genes associated with inflammation and fibrosis processes decreased significantly, and the expression levels of genes related to angiogenesis highly expressed in SNAP group and S-DTMS group, and myocardial rhythm, actin filamentation, and cell activation noticeably increased in both DTMS group and S-DTMS group (Fig. S19). The differential gene volcano map and bubble map analysis of each group also showed that the gene expression level associated with tissue repairing in the S-DTMS group was higher, while the expression associated with inflammation dropped (Fig. 5b). In the early inflammatory stage of the S-DTMS group, S-DTMS hydrogel suture treatment rescued ischemic and dying cardiomyocytes and alleviated cellular damage caused by stress and ischemia-reperfusion. In addition, we studied the number and distribution of M1 and M2 macrophages on day 7 (Fig. 5c–e). We found that there were much more M1 macrophages which associated with inflammation in the MI group (3 times more cells than SNAP group). More M2 macrophages, which associated with tissue repair was observed in the SNAP groups, DTMS groups, and S-DTMS groups, especially the S-DTMS group (13 times more cells than MI group). We then examined the inflammatory factor levels in rat tissue homogenates in the early stages of myocardial infarction. The results of IL-6 and TNF-α suggested that the S-DTMS group had lower levels of inflammation, compared to the MI groups, SNAP groups and DTMS groups (Fig. 5f, g). Finally, the immunofluorescence staining indicated that the fibrosis of the myocardial infarction area in the S-DTMS group was lighter than that in the MI group, and it had a larger area of surviving myocardial tissue (Fig. S20a-c).

### S-DTMS effectively promoting vascular regeneration

We evaluated angiogenesis by quantifying the regenerated capillaries versus myogenic arterioles after treatment. Both micro-fil casting and Micro-CT coronary angiography after 1 month showed higher capillary density in the DTMS groups, SNAP groups and especially S-DTMS groups (Fig. 5h). Notably, the vascularity in the S-DTMS group showed a trend of compensatory hyperplasia of the collateral circulation, and branched vessels covering the LV surface, which explaining the higher LVEF and near-normal myocardial tissue structure in the S-DTMS group. Immunofluorescence staining result showed that, compared with MI group (9.97 ± 6.10/mm^2^), the DTMS group (32.74 ± 13.78/mm^2^), SNAP group (60.75 ± 14.81/mm^2^) and the S-DTMS (68.94 ± 20.90/mm^2^) had a higher density of CD31^+^ capillaries, close to normal myocardial tissue (100.96 ± 19.71/mm^2^) (Fig. 5i, j). Capillary is beneficial for effective survival of myocardium and recovery of cardiac function. Interestingly, although the SNAP group had the most abundant capillary density, the muscular arterioles were less abundant than those in the MI and DTMS groups. This may be due to NO mainly inducing the budding and maturation of microvessels without inducing the proliferation of smooth muscle.

### S-DTMS improving cardiac function and ventricular remodeling after myocardial infarction in pigs

**Fig. 6:**
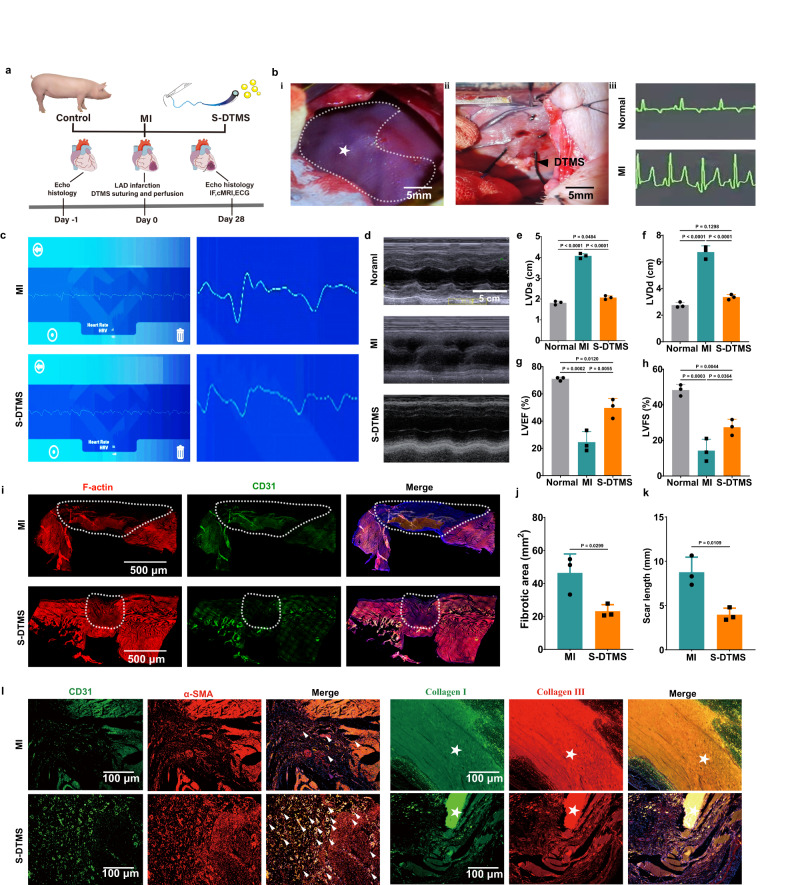
S-DTMS treating porcine myocardial infarction. **a** Scheme of the treatment of myocardial infarction in minipigs. **b** (**i**) Ligated the Left Anterior descending artery (LAD). (**ii**) The myocardium was ischemic and S-DTMS was sutured in the ischemic area. Differences in the ECG of pigs before and after ligation of LAD. *N* = 3 biologically independent replicates. **c** One month later, the ECG of pigs was measured by the Bluetooth ECG connected with the S-DTMS. *N* = 3 biologically independent replicates. **d** Representative images of M-mode ultrasound of pigs after one month. **e**–**h** After 1 month, LVDd **e**, LVDs **f**, LVEF **g**, and LVFS **h** of pigs were assessed by echocardiography. *N* = 3 biologically independent replicates. one-way ANOVA with multiple comparison tests. All values are presented as mean ± SD. **i** One month later, the myocardial tissue’s immunofluorescence staining of SNAP and MI group. From left to right, F-actin^+^ (red), CD31^+^ (green), merge. **j**, **k** Fibrosis coverage area **j** and absolute length of cardiac short axis scar. **k** Two-tailed unpaired Student’s t-test. All values are presented as mean ± SD. *N* = 3 biologically independent replicates. **k** After 1 month, immunofluorescence staining of myocardial tissue in SNAP and MI groups. CD31, α-SMA; Collagen I, CollagenIII. Arrows and asterisks, respectively indicate the areas and tissues with positive immunofluorescence staining. *N* = 3 biologically independent replicates.

We further evaluated DTMS in the MI minipig model (Fig. 6a, b). After MI occurred, DTMS was surgically transplanted into injured myocardial tissue. SANP was pump into infarcted tissue by micropump and DTMS. One month later, our suture still could transform the S-T-segment-elevation ECG signal to the mobile phone via Bluetooth module (Fig. 6c, Supplementary Movie 5). Both MI and S-DTMS groups showed S-T elevation which is typical characteristic of MI. We used echocardiography to study the cardiac diastolic and systolic functions via (Fig. 6d). Left ventricular diastolic dysfunction (LVDd) and left ventricular systolic dimension (LVDs) showed almost two times dilation in the MI group (LVDd:6.74 ± 0.38 cm, LVDs:4.07 ± 0.29 cm) compared with the S-DTMS group (LVDd:3.35 ± 0.15 cm, LVDs:2.07 ± 0.17 cm) (Fig. 6e, f). The Left ventricular ejection fractions (LVEF) significantly decreased in the MI group (24.54 ± 6.30%); however, the S-DTMS group (49.67 ± 5.76%) was close to normal. Left ventricular fractional shortening (LVFs) suggests a decompensated status in the MI group (14.28 ± 5.21%) happened. But S-DTMS (LVFS:27.43 ± 3.63%) had noticeable improvement with our smart suture and drug delivery (Fig. 6g, h). Our DTMS led to less pathological ventricular remodeling and preserved systole and diastolic functions, and blood could be pumped out of the heart efficiently. Immunofluorescence in collagen I and III, α smooth muscle actin, and CD31 staining also supported this conclusion. The scar tissue in the MI group was 1.8 times wide and 2 times in a large area compared to the S-DTMS group (Fig. 6i–k). We found S-DTMS also promoted more blood vessel formation in pigs and reduced collagen I and collagen III deposition (Fig. 6l).

**Fig. 7:**
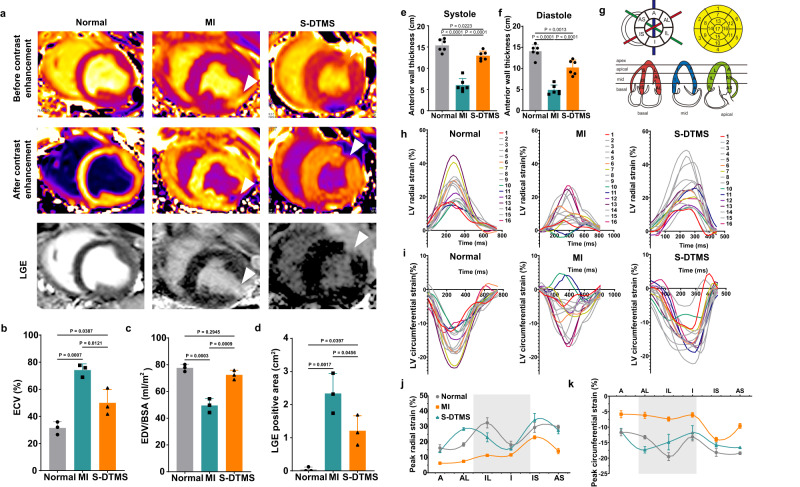
Evaluation of cardiac function after myocardial infarction in pigs by cMRI. **a** CMRI of pigs after 1 month. The mapping image of the pig heart before contrast enhancement, and the mapping image after contrast enhancement at the middle horizontal plane. Short axis myocardial delayed enhancement sequence (LGE). The arrow points to the infarct area. **b** Quantitative analysis of extracellular volume time-sharing. **c** Quantitative analysis of EDV/BSA. **d** Quantitative analysis of LGE positive area. **e, f** Systolic and diastolic LV wall thickness based on myocardial MRI imaging. **g** AHA cardiac segment grading criteria. **h**, **i** In the heart cycle, the radial **h** and circumferential strain (**i**) of all segments of the left ventricle. **j**, **k** Radial strain **j** and circumferential strain **k** peaks in different segments of the middle segment of the left ventricle. **b**–**f** One-way ANOVA with multiple comparison tests. All values are presented as mean ± SD. *N* = 3 biologically independent replicates.

We then applied cMRI to characterize the left ventricular tissue (Fig. 7a). We observed transmural MI happened extensively in the left ventricle in the MI group, which has a risk of heart rupture. In contrast, it didn’t show noticeably in the S-DTMS group. Late gadolinium enhancement (LGE) sequence showed that the contrast medium had a substantial decrease in the infarcted area of the MI group due to the loss of normal cellular structure in the scar area, compared with the S-DTMS group. A larger LGE-positive area in the MI group (1.9 times that of the S-DTMS group) suggested that the majority of cardiomyocytes were lost (Fig. 7d). We then used extracellular volume time-sharing (ECV) to calculate the volume range occupied by non-cardiomyocyte components in myocardial tissue, i.e., the proportion of the extracellular matrix. 74.14 ± 3.79% of ECV in the MI group had 1.5 folds of the S-DTMS group (48.34 ± 8.12%). It showed DTMS extenuated the deposition of extracellular matrix MI area by reducing fibrosis to alleviate the limitation of diastole and systole function of the heart (Fig. 7b, c). We also observed the ventricular wall thinning in the MI group with 0.48 times of the S-DTMS group (Fig. 7e, f). Further, we quantitatively evaluated segmental cardio tissue strain via cardiovascular magnetic resonance feature tracking technology (CMR-FT). All heart segments are divided and quantitatively tested according to AHA standards (Fig. 7g, FigS. 21-23). During a cardiac cycle, a time-series-based evaluation of myocardial segments suggested an overall decrease in radial and circumferential strain in the MI group (Fig. 7h, i). Peak strain parameters also supported this conclusion (Fig. 7j, k). The results of CMR-FT supported more severe cardiac adverse events in the MI group, while DTMS could reduce the risk of cardiac events.

## Discussion

Multifunctional cardio patches for MI are not able to conduct the integration of diagnosis-therapy-monitoring. Here, we report a microchanneled hydrogel suture for heart repair. The elasticity, low friction, and stiffness of the hydrogel sutures ensure that it can maintain good mechanical properties to adapt to the tension of the wound edema, and after the edema subsides, the original tension can be restored. Moderate elasticity avoids the potential risk of tissue ischemia and necrosis caused by sutures. At the same time, the micro-flow channel of the hydrogel suture also provides a two-way drug delivery and body fluid signal acquisition pathway. By easily adjusting the composition of the hydrogel sutures, the hydrogel is endowed with additional functions, such as photothermal effects and force luminescence. Thus, while the DTMS with microfluidic tract has capillary phenomena, the photothermal effect of DTMS provides a method to kill pathogens extensively based on physical methods, avoiding the potential risk of infection caused by DTMS allowing fluid flow through the tissue barrier. And force-induced luminescence hydrogel sutures can convert stress stimuli into an optical signal and can be used as potential novel sensors to detect life signals such as motion, respiration, and pulse^32,34^

Conductive hydrogels carrying microfluidic channels have tunable dimensions and biosafety, drug-controlled release, and sensors in the deep diseased tissue. In rats, DTMS sutures can detect the muscle action potential and identify intestinal paralysis, thrombosis, and myocardial infarction. The difference between DTMS and the components of biosensors such as patches is that DTMS can be precisely combined with the tissue, reducing the noise caused by large-area electrode patches, and avoiding signal interference caused by poor binding, and is more sensitive and accurately reflects the electrophysiological characteristics of the tissue. Good electrical conductivity and a wide range of application scenarios provide evidence that DTMS can be used as a hydrogel electrode with good biological affinity, with great monitoring and diagnostic potential for deep complex soft tissues and organs. As far as we know, this work is the first known application of drug-delivery sutures in the treatment of myocardial infarction. In both rat and porcine MI models, DTMS restored cardiac function by providing a favorable mechanical environment for the myocardium and delivered to accelerate the repair process^35,36^. Interestingly, although nitric oxide prodrugs N-Acetyl-3-Nitrososulfanyl-Valine (SNAP) have already been proved that it could treat MI via reducing the load on the ventricular muscles. The SNAP group did not significantly save the survival rate and heart function of MI rats. This may be related to its excessively short half-life. However, our results suggest that DTMS and the nitric oxide prodrugs can inhibit the inflammatory response and recruit repair-related macrophages in the early stages of trauma, reducing oxidative stress caused by inflammation and ischemia, and thereby promoting tissue recovery. In the pig myocardial infarction model, DTMS effectively reduced the area of transmural myocardial infarction and improved abnormal ventricular remodeling to save cardiac function. This indicates that DTMS plays a role in diagnosing, treating, and reducing the incidence of malignant cardiovascular complications in the treatment of cardiac ischemic diseases.

The DTMS can be compact and flexibly used to judge the occurrence and prognosis of the disease, and to deliver the drugs into the lesion timely and accurately. It has important significance for the diagnosis of the condition outcome after treatment.

## Methods

### Preparation of hydrogel sutures and conductive hydrogel sutures

The PVA (Alading, Mw-20,500, P119362-50g) (100 mg/ml) was dissolved in deionized water at 90°C, stirring at a constant speed until completely dissolved evenly into a transparent solution^22^. The hydrogel sutures and conductive hydrogel sutures are made in the same way (Polypyrrole (MACKLIN, P871996-5g) and the PVA solution mass are 1:9). The custom stepper motor enables the hydrogel to continuously and evenly cover the surface of different sizes of nylon fiber mold (outer diameter~ 50 μm, 100 μm, 200 μm, 300 μm, 1 mm, etc.). To avoid electromagnetic interference from the tissue environment, one end of the conductive suture was coated with a thin layer of insulating PVA hydrogel in the same way. After drying overnight at room temperature, the fully dried hydrogel was immersed in 6 mol of sodium hydroxide solution for 20 min. The hydrogels were subsequently transferred to ultrapure water and soaked overnight. Stretch the end of the hydrogel and the hydrogel was gently removed from the mold to become the hydrogel suture.

### Characterization of scanning electron microscope

After freeze-drying for 48 h, the hydrogel sutures were adhered to the surface of the conductive adhesive base for gold injection. Next, the hydrogel sutures and the conductive hydrogel sutures were subsequently characterized using a scanning electron microscope (ZEISS, Crossbeam 340).

### Mechanical properties

To test the mechanical properties of the sutures, we used a mechanical property tester (QT-6201S) for the stress-strain, fracture strength, and knot strength, and cyclic tensile tests. All the sutures including DTMS (ID = 50 μm, OD = 80 and 200 μm), silk suture® (diameter = 200 μm and 1 mm, JUNSHENG), nylon suture® (diameter = 100 μm, LINGQIAO) were cut into 2 cm segments and mounted to the fixture. The fixture performs uniaxial motion at a constant speed of 100/min. The nominal tensile strain (ε) was defined as the change in the length (Δl) divided by the original length (l_0_) of the specimen.

1 $${{{{{\rm{\varepsilon }}}}}}=\Delta {{{{{\rm{l}}}}}} \, / \, {{{{{{\rm{l}}}}}}}_{0}$$

The nominal stress (σ) was obtained by dividing the force (F) by the original cross-sectional area (A_0_) of the specimen:

2 $${{{{{\rm{\sigma }}}}}}={{{{{\rm{F}}}}}}/{{{{{{\rm{A}}}}}}}_{0}$$

The tensile strength and breaking elongation of the hydrogels were obtained from the stress-strain curves. The toughness (*T*, MJ m^-3^) of the hydrogel was calculated through integrating the area below the tensile stress-strain curve, and the calculation equation is as follows:

3 $${T}={\int }_{{\ \!\!\!\sum}=0}^ {\!\sum=\varepsilon b}{\sigma }_{b}{{{{{\rm{d}}}}}}\varepsilon$$

where *σ*_*b*_ and *εb* are the tensile strength and corresponding the fracture elongation, respectively.

The cyclic loading-unloading tensile test was performed at different strains (20%, 40%, 80%, 100%, 120%, 150%, 200%, 250%, and 300%,) to investigate the energy dissipated mechanism of the hydrogels. The dissipated energy (hysteresis, *U*_*hys*_) was calculated from the area of the hysteresis loop between the loading-unloading curves according to the following equation:

4 $${U}_{{hys}}=\int _{\varepsilon=0}^{\varepsilon={\varepsilon }_{x}}\left({\sigma }_{{load}}-{\sigma }_{{unload}}\right){{{{{\rm{d}}}}}}\varepsilon$$

The data was processed in GraphPad Prism8.

### Electrical conductivity test

Electrical conductivity was measured by two-probe method (Victor 4092 C), bias voltage over the range from -0.5 to 0.5 V was applied ends of DTMS (OD=100μm, ID=80μm, length=10 mm). Different concentrations of PPy (DTMS containing 1%, 5%, 10% PPy) was tested. All electrical conductivity values reflect the mean of at least five samples for each condition.

### Friction force test

The test of friction is to measure the tissue resistance using a mechanical property tester (QT-6201S). Tissues were fixed on the bottom fixture. All the suture (DTMS and PRIS with ID = 80 μm and OD = 200 μm), silk suture with diameter = 200 μm) passed through the tissue with the same depth and length. After the sutures passed horizontally through the heart muscle, skeletal muscle and liver of the rat, the sutures were fixed on the upper fixture. Then the upper fixture moved with a constant speed of 100 mm/min. The static friction and sliding friction between the tissue and the sutures was measured, and the data was processed in GraphPad Prism8.

### Suture tissue damage test

SD rats (Male, 230–250 g) were anesthetized and mechanically ventilated by inhalation of isoflurane (RWD, R510-22-10). The DTMS and 5-0 silk sutures were threaded through the rat’s back tissue and surgically knotted. After 7 days, the tissue was fixed and stained with HE, and the tissue homogenate was extracted and used for ELISA detection (Invitrogen, A43656; EMMPO).

### The perfusion function test of micro channeled hydrogel suture

In the in-vitro test, the microchannel of suture (OD = 200 μm, ID = 80 μm) was connected to the 1 ml syringe with 34 G needle, and the FITC solution perfused through the microfluidic channel of the suture. In the in vivo experiment, after the suture extended from the chest through the back skin, the microchannel of the suture was connected to the insulin injection pump (MICROTECH MEDICAL, HRN-S-60). So that the Iohexol (MACKLIN, I837876) could be delivered quantitatively to the sutured site. In order for the Iohexol solution could pass smoothly through the microfluidic channel with the suture knotted, on the basis of intermittent or continuous suturing, additional continuous suturing allow the microfluidic channel to maintain its integrity. Micropores are reserved at the ends of the sutures to allow fluid to pass smoothly under pressure.

In order to test the potential of DTMS to extract liquid, we used DTMS to anastomose the back tissue and connected one end of the DTMS to a clinical dynamic blood glucose meter (YvYue, GU200) to record 24 h glucose levels and peripheral blood glucose every 2 h (Contour Plus).

### Diffusion test

To mimic the tissue environment, we made the gelatin tissue and the sutures prefilled with red dye (1 cm long, 200 μm outer diameter and 80 μm inner diameter) were embedded into the gelatin. The area of dye diffusion in the gelatin tissue was recorded by camera, measured, and analyzed by imageJ.

### Preparation and characterization of force-luminescent sutures

Force luminescent sutures were made in the same way as DTMS sutures, 10% PVA solution to evenly covered the surface of the nylon fiber mold, after complete drying, the PDMS containing 10% ZnS:Cu (Molbase, 68611-70-1) solid powder was applied evenly to the outer membrane of the hydrogel^31^, after 60 °C crosslinking for 2 h, 10% PVA solution coated the surface again. After drying overnight, the hydrogel was immersed in 6 mol of sodium hydroxide solution (Aladdin, 1310-73-2) for 20 min. The hydrogels were subsequently transferred to ultrapure water and soaked overnight. They were slowly divesting the hydrogel off the nylon fiber surface to get the force-luminescent suture. The SEM characterization was the same as that for the PVA sutures. Mechanoluminescence properties are achieved by mechanical property tester (QT-6201S). For easy observation, we cut the suture with 1.5 mm outer diameter into 2 cm segments. The suture was fixed on the fixture and performed uniaxial cyclic stretching. (Strain: 0-100%; Frequencies: 0.5 Hz, 1 Hz, 5 Hz and 10 Hz).

### Characterization of the photothermal effect of hydrogel sutures

The photothermal effects of the sutures are characterized by infrared imaging. The thermal distribution of the heated sutures starting from room temperature was imaged with 808 nm infrared light and recorded by thermal imager. In vivo tests, after treating MI, DTMS elicited from the intercostal space and placed subcutaneously. The DMTS under skin was was warmed using 808 nm near-infrared light, the laser was 15 cm away from the skin, and the suture heat distribution was measured with a thermal imager (HIKMICRO, HM-TPH21Pro-3AQF).

The photothermal bactericidal ability of the sutures was evaluated in the same way. After being cultured, Staphylococcus aureus was transferred to EP tube, and was irradiated by 808 nm near-infrared light with PRIS, DTMS and 5-0 silk thread (distance: 15 cm, power: 3 W, time: 15 min). The thermal imager (HIKMICRO, HM-TPH21Pro-3AQF) records the temperature of all groups. Then, transfer the bacterial solution of each group to 96-well plate, continue to culture, and detect the value of OD600 at a fixed time.

### Biosafety evaluation

The biosafety of sutures was performed by incubation of human umbilical vein endothelial cells (HUVEC) and rat myocardial cell lines (H9C2) with hydrogel sutures in transwell chamber (6.5 mm diameter, Corning). After 48 hours co-incubated with sutures, cells were stained for live-dead cells with Calcein AM and propidium iodide (Dowobio, DW2065), and cell activity was examined by MTT (Invitrogen, V13154). The biosafety performance of SNAP was performed by treating SNAP and 100 μM hydrogen peroxide with rat myocardial cell line (H9C2) for 48 h, followed by detection of cell activity by the MTT assay. All the result analyzed by GraphPad Prism8.

### Animal experiments

SPF-grade SD rats (Male, 230-250 g) were purchased from the experimental animal center of the Third Military Medical University (license No. scxk (Yu) 2017-0002). Mini pigs (Male, 1 year old, 30-40 kg) were purchased from the experimental animal center of the Third Military Medical University (license No. scxk (Yu) 2017-0002). All rats were raised in a single cage in the SPF animal room, and all animal experiments were approved by the animal ethics committee of the Third Military Medical University (approval No.SYXK(Yu) 20170002 approval time: 2020.4.20). All experimental operations were performed in accordance with the guidelines for humane treatment of experimental animals.

### Evaluation of the DTMS’s sensor function

DTMS (length: 1 cm, outer diameter: 100 μm, Inner diameter: 80 μm; outer diameter: 200 μm, inner diameter: 100 μm; 400 μm, inner diameter: 200 μm) loaded on a mechanical property tester (QT-6201S). The fixture performed three kinds of steps (Sin, Square, Triangle), and the motion at a strain from 0 to 150%, while the resistance of the DTMS suture was measured using a digital oscilloscope (Tektronix, 2110-220)^7^. Human motion perception is realized by attaching DTMS to the fingers and knee joints and connecting the digital oscilloscope (Tektronix) to measure the resistance.

For body surface electromyography signals received by the Bluetooth module in different states. DTMS was pasted to the skin of quadriceps, and the DTMS connected with Bluetooth module (BMD 101). In a sitting, standing, walking, jumping and stationary state, the DTMS and Bluetooth module that remaining fixed with the skin receive EMG signals from the body surface. The electronic signal was recorded by computer and analyzed by GraphPad Prism8.

For animal’s surface and deep tissue of the physiological signal detection, after the rats (SD rats, male, 230–250 g) were inhalation isoflurane (RWD, R510-22-10) anesthetized. DTMS was anastomosed respectively on lower limb skeletal muscle muscles, colon and abdominal aorta and heart. To detect bioelectric signal, after tying the knotted and keeping DTMS in relatively stable, the endpoint of DTMS was connected with Bluetooth module (BMD 101). The electronic signal was recorded by computer and analyzed by GraphPad Prism8. To simulate the disease model of muscular soft paralysis, intestinal paralysis, and thrombosis, after recording the initial electrophysiological activity, we performed the lower limb sciatic nerve detachment, colon extrusion and friction, and clamping of the abdominal aorta.

### Rat myocardial infarction model and suture implantation

SD rats (Male, 230–250 g) were anesthetized and mechanically ventilated by inhalation of isoflurane (RWD, R510-22-10), and after opening the chest cavity, the LAD was ligated using an 8-0 polypropylene suture. At 2 h after LAD ligation, rats were randomized into MI, SNAP, DTMS and S-DTMS. For the Shank and SNAP group, DTMS was sutured continuously for 3–4 stitches to cover the infarcted area. One end of the suture extends under the skin of the back, attached to the insulin pump reservoir (MICROTECH MEDICAL, HRN-S-60). After suture implantation, the wound was closed and sterilized, and the shank group was injected with 300 μl of saline (SHIMEN, BR20500) through a reservoir and the S-DTMS group with 300 μl of 1 mM/ml SNAP solution through a reservoir. And the SNAP groups’ infarcted area was injected with 300 μl of 1 mM/ml SNAP solution. Penicillin sodium was administered intramuscularly once per day for 3 days.

### Echocardiographic evaluation of cardiac function in rats

Rats were inhalation-anesthetized with isoflurane at 0 d, 7 d, 14 d, 28 d, 60 d, and 90 d after MI and underwent transthoracic echocardiography of all groups using ultrasound (Esaote, MyLab™). The M mode tracking was recorded in the left ventricular long axis, measuring the percentage LVEF, already in the LVDd and LVDS, and measuring at least 3 cardiac cycles per image.

### The ECG examination for the rats

For the rats MI treatment and ECG detection, DTMS was implemented on the surface of the left ventricular wall. One of the endpoints of the suture was fixed on the heart’s surface, while another endpoint was expanded from the chest and connected with the Bluetooth module (BMD 101). One month later, rats from all groups were monitored by Bluetooth module, and the ECG signals were displayed on mobile device. The data were collected via ThingGear software and processed via GraphPad Prism9. To verify accuracy of the DTMS’s sensing signal, we measured the body surface ECG of rats using an electrophysiology station (iWorx RA 834). The data was collected via RM6240E-V2.5 and processed by GraphPad Prism9.

### PET and PET-CT imaging of the rat hearts

For PET imaging, rats were anesthetized and mechanically ventilated by inhalation of isoflurane, and 18F-FDG with 500 Ci was injected via tail vein injection, after 1 h later, rats were scanned using acquired images from the Trans-PET BioCaliburn 700 system. The PET images were processed using a 3DOSEM. Image analysis was performed in three axial, coronal, and sagittal directions using Carimas software.

### Histological evaluation

On day 0, day 7, and day 30, rats were euthanized and subsequently infused with PBS (Hyclone, SH30258.01). Finally, infused with 4% paraformaldehyde for 20 min and hearts were collected. Serial sections were performed at the ventricular short axis level after paraffin embedding. Paraffin sections were stained with Masson, HE, and the overall area was measured with ImageJ (v1.51) by the ratio of the inner perimeter of the scar region to the circumference of the inner circumference of overall LV wall, and we also measured the tissue thickness of the infarct region using imageJ. For immunofluorescence staining, paraffin sections from all groups at 7d and 30d were incubated overnight with rabbit anti-CD-31 (1: 500, Abcam, ab222783), mouse anti-α-SMA (1:300, Boster, BM0002); rabbit anti-CD-86 (1:300, Abcam, ab239075), mouse anti-CD-163 (1:300, Abcam, ab182422), washed twice in PBS, incubated with the corresponding secondary antibody (Alexa Fluor 488- and Alexa Fluor 568-conjugated antibodies (1:800, Invitrogen, A11001; 1:800, Invitrogen, A11004)) at 37 °C for 1 h, and nuclei were stained with anti-fluorescence quenching tablets (Boster) and sealed. Immunofluorescence-stained sections were visualized using a fluorescence microscope (ZEISS LSM 800), and blood vessel numbers, macrophages numbers, fiber thickness, and area were counted using the ImageJ. At least 3 visual fields were used per sample.

### Rat heart microcirculation perfusion and Micro-CT

Rats were anesthetized with 2% isoflurane and underwent left common carotid artery catheterization with a 22 G Teflon tube, and the tube orifice reached the level of the thoracic aortic sinus. After humanely euthanizing the rat, the coronary arteries were perfused with a pre-configured MicroFil (Flow Tech Inc, MV-122) molded contrast medium until they fully filled the coronary arteries and cardiac capillaries, the common carotid artery was ligated to prevent contrast outflow, and the anesthetized rats were euthanized. Rat carcasses were maintained at 4 °C overnight to facilitate contrast cross-linking, and subsequently enhanced contrast scanning of the rat cardiac microcirculation was performed using a micro-CT (Bruker micro-CT).

### In vivo fluorescence measurements

To verify the perfusable potential of the hydrogel suture microflux in the body, the BSA-DIR was pumped via DTMS’s microchannel and perfused to the rat’s heart surface (100 μL/min). On day 0, day 1, day 3, day 7, day 9, day 11, day 13, and day 15, rats were anesthetized with isoflurane, fluorescent images obtained. On day 15, the dye was re-perfused to verify the ability of DTMS to deliver the drug multiple times. After acquiring images, radiation efficiency was analyzed using automatic circular ROI.

### Bulk RNA sequencing

We extracted the left ventricular wall tissue a week after therapy. Total RNA was extracted, and RNA quality was detected. After constructing the general transcriptome library, sequencing was performed on the Illumina NovaSeq 6000 (Berry Genomics Corporation, Beijing, China). For differentially expressed gene (DEG) analysis, significantly changed genes were selected (fold-change, 1.0 with q value < 0.05) by edgeR v3.3.81. Kyoto Encyclopedia of Genes and Genomes (KEGG) functional enrichment (over representation) of DEGs was analyzed using a R package clusterProfiler v4.4.4.

### Porcine model of MI and hydrogel sutures implantation

Male minipigs (1 year old, 30–40 kg) were randomized into the normal, MI and S-DTMS groups. All pigs were kept in solitary for one month and monitored daily. Before surgery, pigs were intramuscular injected with 10 mg/kg ketamine hydrochloride (Sigma-Aldrich, N-036-1ML) and 1 mg/kg Xylazine Hydrochloride Injection anesthesia. After induction of anesthesia, the pigs were fixed supine on the operating table and ventilated by endotracheal intubation and maintained with 2% isoflurane. After changing the pig to the right lateral decubitus position, the thoracotomy was performed in the fourth and fifth rib spaces, and after expanding the surgical space using a rib opener, the pericardium was cut and suspended with a 5-0 suture to observe the LAD. An acute myocardial infarction model in pigs was constructed by sealing the LAD distal to the second diagonal branch start with a 5-0 polypropylene suture for 10 min, and after determining the surgical position. ECG, respiration, body temperature as well as oxygen saturation were monitored throughout the procedure. Intraoperative ST segment elevation was used as an indicator of acute myocardial infarction. After visual determination of the infarct range, based on the area of ischemic whitening, the infarct area was stitched continuously with a 1 mm diameter hydrogel suture, and the suture area did not exceed the ischemic area. After introducing the suture endpoints to the skin, connect the Bluetooth module (BMD101) and the insulin pump module respectively. After layer closure, myocardial electrophysiology was monitored using a mobile phone and controlled by pump injection of 1 ml of 10 micromolar solution of SNAP into the microfluidic channel. The postoperative procedures were followed with daily intramuscular antibiotics to restore the animals.

### Transthoracic echocardiography of minipigs

Transthoracic echocardiography was performed on small pigs before and 30 days after surgery using a machine (Esaote). For examining anesthetized small pigs in the left lateral horizontal position, dynamic images of LV were captured by transducer, recorded, and analyzed at the long axis level using (type of instrument) M ultrasound, recording LVDD, LVDS, FS, and EF.

### Magnetic resonance detection of minipigs

Cardiac magnetic resonance (CMR) imaging studies were carried out 1month after the induction of MI, using a 3.0-Tesla MRI scanner (Trio, Siemens Healthineers, Erlangen, Germany) equipped with a 12-element phased array surface coil. Shimming and center frequency adjustments were performed prior to CMR imaging to minimize off-resonance artifacts.

A breath-hold, retrospective, electrocardiogram (ECG)-gated balanced steady-state free-precision cine sequence (bSSFP) was used to obtain cine images, including the LV 2- and 4- chamber long-axis and short-axis stack covering the entire LV from base to apex. T1 mapping was performed at the basal, mid and apical slices of the left ventricular short axis using a modified Look-Locker Inversion Recovery (MOLLI) prototype sequence: 5(3)3 and 4(1)3(1)2 acquisition protocol was adopted for native and post-contrast T1-mapping, respectively. (bSSFP readout mode, field of view, 400 × 300 mm^2^; matrix, 256 × 166; TR/TE, 301.7/1.09 ms; flip angle, 35 degrees; and 6 mm thickness). Post-contrast T1-mapping and standard LGE imaging was performed 10 min after injection of a 0.2 mmol/kg bolus of gadoteric acid meglumine (Dotarem, Guerbet, BP7400, F95943, Roissy CdG Cedex, France). LGE imaging was performed with a phase-sensitive inversion recovery sequence (PSIR) with the following parameters: FOV 400 × 300 mm^2^, matrix 256 × 166, TR/TE 301.7/1.09 ms, flip angle 35 degrees, 6 mm thickness.

All the cine and T1 map images were analyzed offline using the cvi42 software (Circle Cardiovascular Imaging Inc., Calgary, Alberta, Canada). Endo- and epicardial contours on the cine images were manually traced to obtain the LV end-diastolic volume (EDV), end-systolic volume (ESV), stroke volume (SV), ejection fraction (EF), cardiac index (CI), and myocardial mass with papillary muscle included in the LV lumen. Myocardial feature tracking analysis was performed on the four-chamber long-axis cine images to acquire LV global longitudinal strain.

For ECV measurements, an ROI was manually selected within the infarcted myocardium and another ROI in the LV cavity to obtain myocardial and blood T1 values. The ECV was calculated from native and post-contrast T1 of myocardium and blood and HCT, using the published formula^37^. The ECV values of the remote myocardium were measured simultaneously as a reference.

### Morphological and histological analysis of the minipig samples

After the minipigs were euthanized, the heart was rapidly excised and fixed with 4% paraformaldehyde (Biosharp, BL539A). After 48 h, the hearts were sectioned from the base to the heart apex. After dewaxing the paraffin sections of pig hearts at 10 μm thickness, hematoxylin eosin and Masson staining was performed according to rat histological staining and immunofluorescence staining. For immunofluorescence, the sample antigen was repaired after dewaxing, treated with rabbit anti-CD-31 (1:500, Abcam, ab222783), mouse anti-α-SMA (1:300, Boster, BM0002); rabbit anti-collagen I (1:500, Abcam, ab138492), mouse anti-collagen III (1:500, Abcam, ab184993); 4 °C overnight, then washed with PBS for 3 times, incubated with secondary antibody (Alexa Fluor 488- and Alexa Fluor 568-conjugated antibodies (1:800, Invitrogen, A11001; 1:800, Invitrogen, A11004)) for 2 h at 37 °C, and sealed with anti-fluorescence quenched tablets containing DAPI. Pictures were observed and collected using a fluorescence microscope (ZEISS LSM 800).

### Statistical analysis

Results are provided as means ± the standard error. All data are from at least three (in vitro and in vivo) independent experiments. Comparisons between the two groups were performed using a two-tailed unpaired Student’s t-test. Multiple comparisons were performed using Ordinary one-way ANOVA and Tukey’s multiple comparisons test. All statistical tests were two-sided, and significance was assigned at *P*  <  0.05. All the data was statistically processed by GraphPad Prism9.

### Reporting summary

Further information on research design is available in the Nature Portfolio Reporting Summary linked to this article.

### Supplementary information

Supplementary Information

Peer Review File

Description of Additional Supplementary Files

Supplementary MovieS1

Supplementary MovieS2

Supplementary MovieS3

Supplementary MovieS4

Supplementary MovieS5

Reporting Summary

### Source data

Source Data

## Data Availability

Source data are available for graphs plotted in Fig. 1–7 and Supplementary Figs. S1–23 and provided as a Source Data file. All raw sequencing data can be found at the NCBI Sequence Read Archive (accession number: GSE252825). All other data are available from the corresponding author upon request. Source data are provided with this paper.
