# Supplementary material for: Two way workable microchanneled hydrogel suture to diagnose, treat and monitor the infarcted heart
Source: Nat Commun. 2024 Jan 29;15:864. doi: 10.1038/s41467-024-45144-y (PMC10824767; doi:10.1038/s41467-024-45144-y)
Supplement: Supplementary file 1 — Supplementary Information [file 41467_2024_45144_MOESM1_ESM.pdf]

Supplementary Information for:

Two way workable microchanneled hydrogel suture to diagnose, treat and monitor the infarcted heart

Table S1

Name	Material	Advantage	Disadvantage	Tensile strength	Absorption rate	Application
**Perma-Hand®**	Silk	Ease of handling,Knot security,Pliability	Low tensile strength, High coefficient of friction, Capillarity, Tissue reactivity	80 – 100% strength lost by 6 months	1 – 2 years	Mucosal surfaces, intertriginous areas,cardiovascular, ophthalmic and neurologicalprocedures
**Vicryl**	Polyglycolic acid and polylactic--acid copolymer 910	Certain anti-inflammatory effect, rapid degradation	Strength and tension fade quickly	50% strength lost by 3 weeks	60-120 days	Soft tissue suture and ligation, including ophthalmology, general surgery, obstetrics and Gynecology, orthopaedic surgery
**Dexon**	Polyglycolic acid	High tensile strength, Excellent knot security	High tissue drag	80% lost at 14 days	60 – 90 days	Caesarean section, hernia repair and in situations where longer wound support is required
**PDSⅡ®**	Polydioxanone	Low tissue reactivity and easily pass through tissue	Poor knot security	Approximately 14% remains at 8 weeks	180 days	Used in areas where longer wound support is required
**Mersilene® Ethibond Excel®**	Polyester	High tensile strength, Good handling	Highest tissue reaction than any other synthetic suture	No appreciable loss of tensile strength	Non-absorbable	Mucosal surfaces, prosthetic implantations,face lifts, general surgery and cardiovascular surgeries
**Prolene®**	Polypropylene	Easily pass through tissues, Minimal host response	High memory poor knot security and lack of elasticity	Infinite	Non-absorbable	Skin closure, including use in cardiovascular,ophthalmic procedures and neurological procedures, subcuticular skin closure
**Ethilonl® Nurolonl®**	Nylon	Low tissue reaction	Poor handling and knot security	15 – 20% strength lost per year	15 – 20% degrades per year	Plastic surgery, neurosurgery and general surgical use

Table S1. Characteristics of commercial surgical sutures

Table S2

	Suture design	mechanical property	Drug loading and release function	Monitoring function	Application scenario	Other functions
DRESS[1]	Polyurethane multifilament conductive fiber core embedded with Ag nanoparticles and poly (vinyl alcohol) (PVA)/poly (N-isopropylacrylamide) (PNIPAm) thermosensitive polymer shell containing drugs	Diameter=600 µm, UTS=75 MPa	Yes. Only hydrophilic drugs can be released at a controlled time and rate by heating the thermosensitive PVA-PNIPAm shell	No	Skin, Achilles tendon	No
LATIS[2]	Incorporation of gold nanorods into collagen fibers	Diameter=70-99 µm, UTS= 5 - 6 Mpa	No	No	Skin	Promote skin wound healing and mechanical strength recovery through light activation
WiSe[3]	Conductive polymer poly (3,4-ethylenedioxythiophene) - poly (styrene sulfonate) (PEDOT: PSS) functional medical grade multifilament suture and micro-electronic pledget for generating incident signal	Diameter=350-400 µm, 28% deformation =320 Mpa	No	Yes. Monitoring the physical and chemical status of the deep surgical site through the capacitive sensor operated by radio frequency identification	Deep surgical wounds	No
BSS[4]	The core fiber is coated with three layers of silk fibroin mixed with different chemical and biological admixtures to form a multi-layer structure "core-shell"	Diameter=120 µm, UTS =450 Mpa	Yes. The release rate of GM-CSF can be accurately controlled by customizing the biodegradation rate of silk shell, and the release rate of GM-CSF can also be accelerated by applying voltage to BSS	Yes.The silk protein coating is functionalized with carbon nanotube dopant, which makes the suture conductive and electrically resistant, and is used to measure the wound stress and monitor the healing process	Skin	Measure the tension of suture tissue and promote wound healing
TGS[5]	Dual network multi-functional tough gel sheath combined with commercial surgical suture	Diameter= 300-340 µm, 2%deformation =3 Gpa	Yes. Load small molecule drugs, and regulate drug release by changing drug concentration or thickness of hydrogel sheath	Yes. PH sensing beads are loaded into TGS to convert pH signals into color changes visible to the naked eye	Tendon, Skin	NIR fluorescence imaging for precise positioning and visualization of suture
BHF[6]	Bacterial cellulose hydrogel based on three-dimensional cellulose nanofiber network	Diameter=500 µm, UTS =90 Mpa	No	No	Skin	No
**DTMS**	Polyvinyl alcohol hydrogel with conductive polypyrrole (Ppy) and microchannel structure	Diameter=80-400 µm, UTS= 2000 Mpa	Yes. Multiple times and on-demand drug supply through micro-channel	Yes. Through the conductivity of polypyrrole (Ppy) and Bluetooth transmission, the monitoring of various physiological signals is realized	Muscle, skin and other tissues	Extract tissue fluid around deep wounds;

Table S2. A summary and comparison of sutures' designs

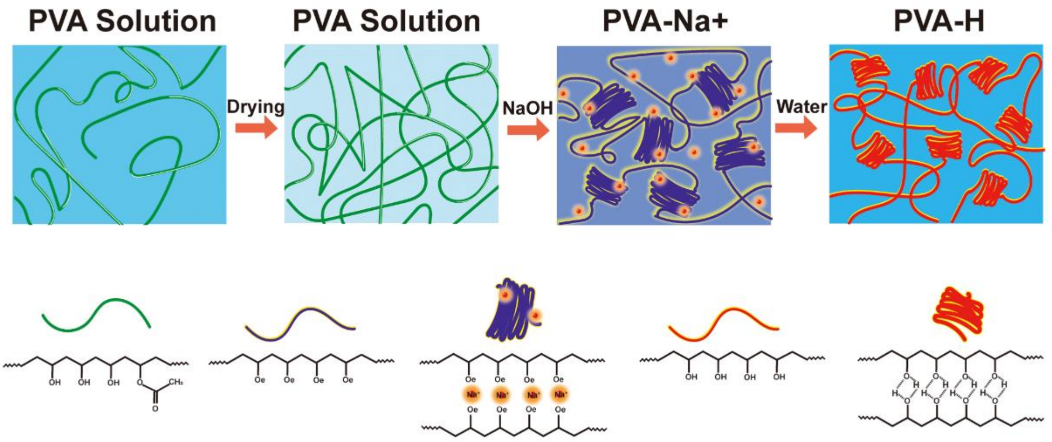

Figure S1. Schematic diagram of PVA preparation and crosslinking: PVA Na +: PVA soaked in NaOH solution; PVA-H: crosslinked PVA hydrogel.

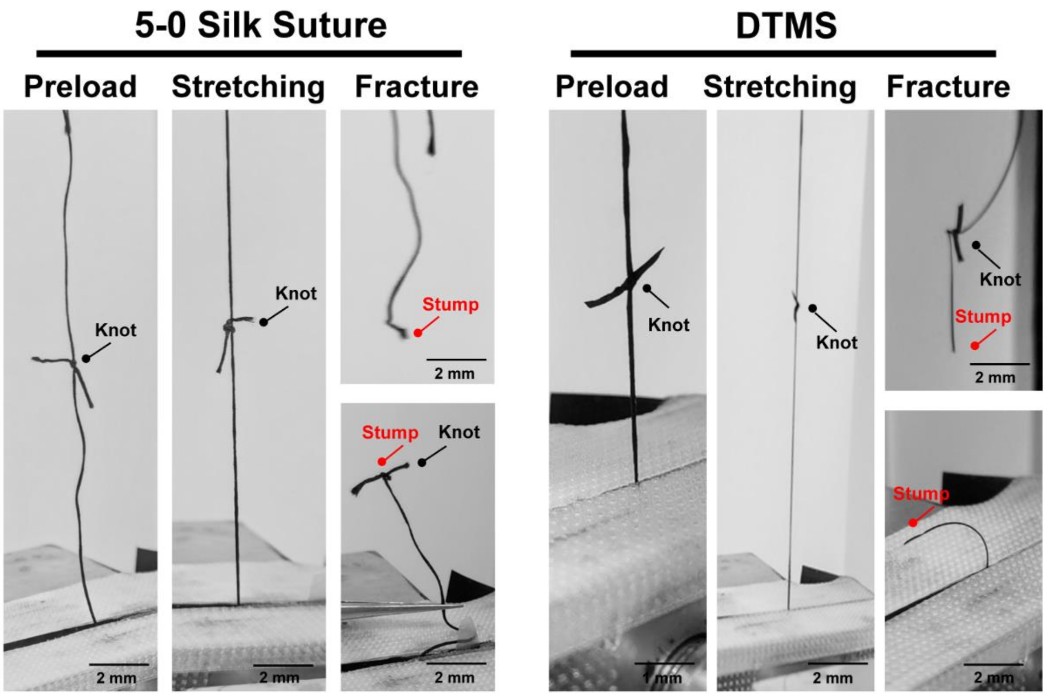

Figure S2. DTMS hydrogel suture and silk suture's knotting test. DTMS (Outer diameter: 200 μm. Inner diameter: 80 μm, length: 5cm.) and 5-0 silk suture (Diameter: 200 μm). After tying a square knot at both ends of the same suture, used a tensile machine to stretch until the suture breaks. The DTMS hydrogel suture line avoids excessive stress on the knot and breaks, due to uniform conductive stress; The stress of the silk suture causes the break at the knot.

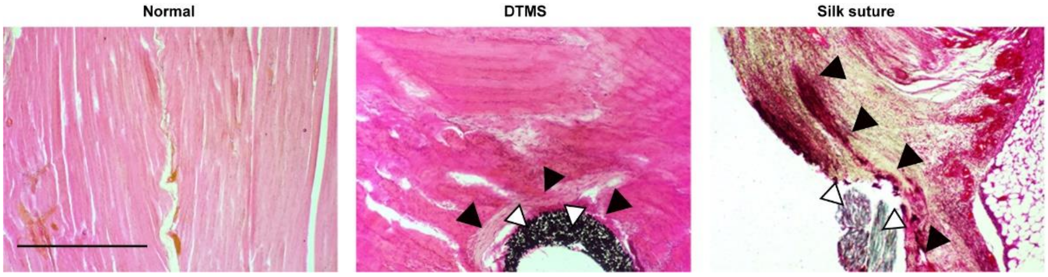

Figure S3. Suture tissue damage test. The DTMS and 5-0 silk suture were respectively threaded through the rat's back tissue and surgically knotted. After 7 days, the tissue was fixed and stained with HE. White arrows indicate DTMS and silk thread respectively. Black arrows indicate areas of tissue damage, inflammation, and necrosis. Bar = 200μm.

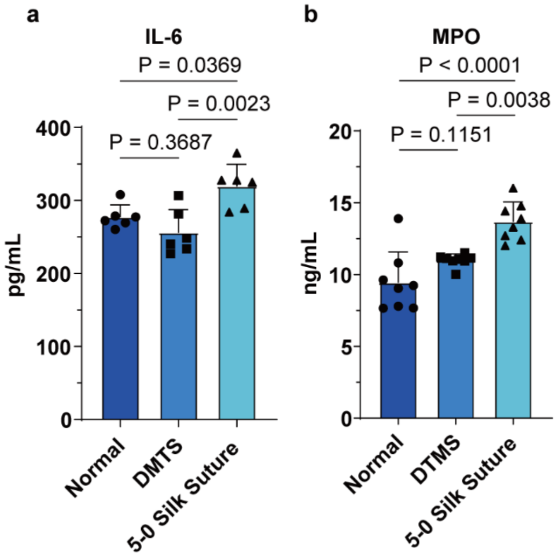

Figure S4. Suture tissue damage test. The DTMS and 5-0 silk suture were respectively threaded through the rat's back tissue and surgically knotted. After 7 days, the tissue homogenate was extracted and used for ELISA detection. a. IL-6, One-way ANOVA with multiple comparison tests. All values are presented as mean ± SD. N=6 biologically independent replicates. b. MPO, One-way ANOVA with multiple comparison tests. All values are presented as mean ± SD. N=8 biologically independent replicates.

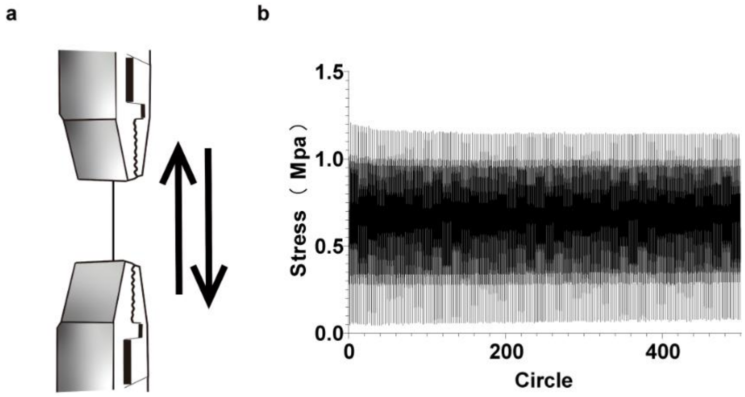

Figure S5. DTMS hydrogel suture cycles: a DTMS motion pattern for cyclic stretching. **b** DTMS cyclic tensile curve. DTMS (Outer diameter: 200μm, inner diameter: 80μm, length: 5cm), tensile

strain: 5%, 0.5Hz, cycle times: 500.

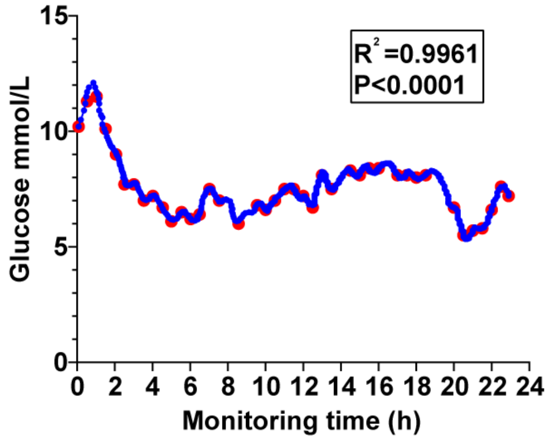

Figure S6. Glucose levels in interstitial fluid extracted by DTMS. Blue dots represent the 24-hour interstitial fluid concentration in deep tissues collected by DTMS, and red represent the peripheral blood glucose concentration collected by blood-glucose meter.

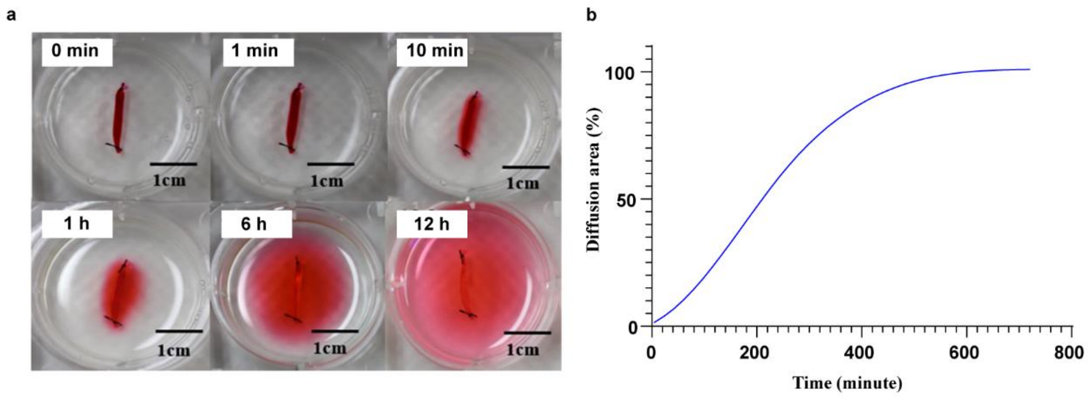

Figure S7. DTMS's small molecule diffusion test: a DTMS (Outer diameter: 1mm, inner diameter: 800μm, length 1cm) was prefilled with red dye and placed in 20% gelatin at room temperature. **b** The diffusion area of small molecule dye. Bar=1cm.

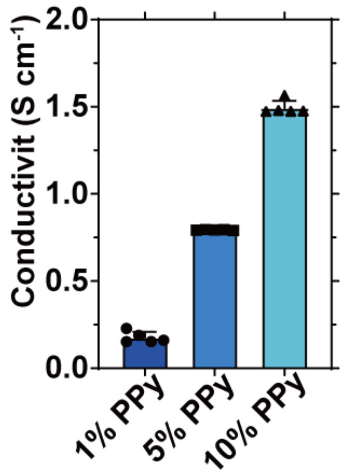

Figure S8. Conductivity of DTMS: DTMS containing 1%, 5%, 10% PPy. All values are presented as mean ± SD, **n=5** independent replicates.

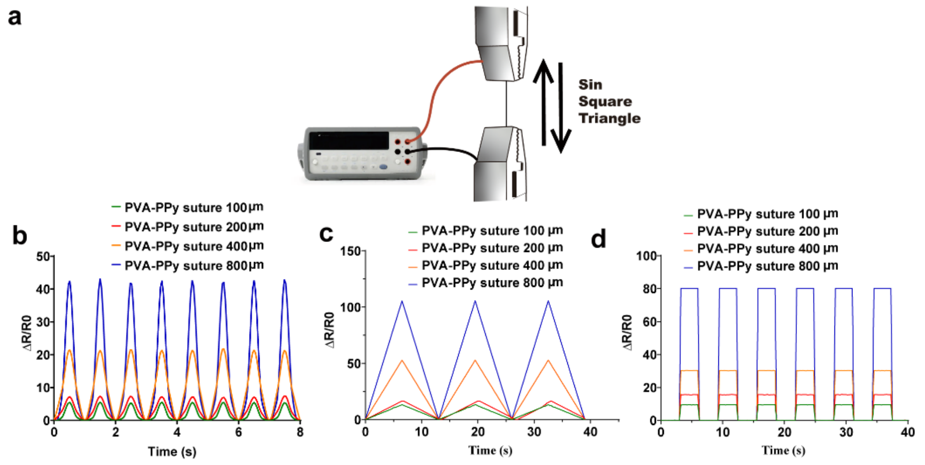

Figure S9. Resistance change of DTMS with different diameters under stretching: a DTMS motion pattern for cyclic stretching. **b** Sin. **c** Square. **d** Triangle. DTMS (Green, outer diameter: 100μm, Inner diameter: 80μm), DTMS (Red, outer diameter: 200μm, inner diameter: 100μm), DTMS (Orange, outer diameter: 400μm, inner diameter: 200μm), DTMS (Blue, outer diameter: 800μm. inner diameter: 400μm), All sutures were 1cm long, strain from 0%-150%.

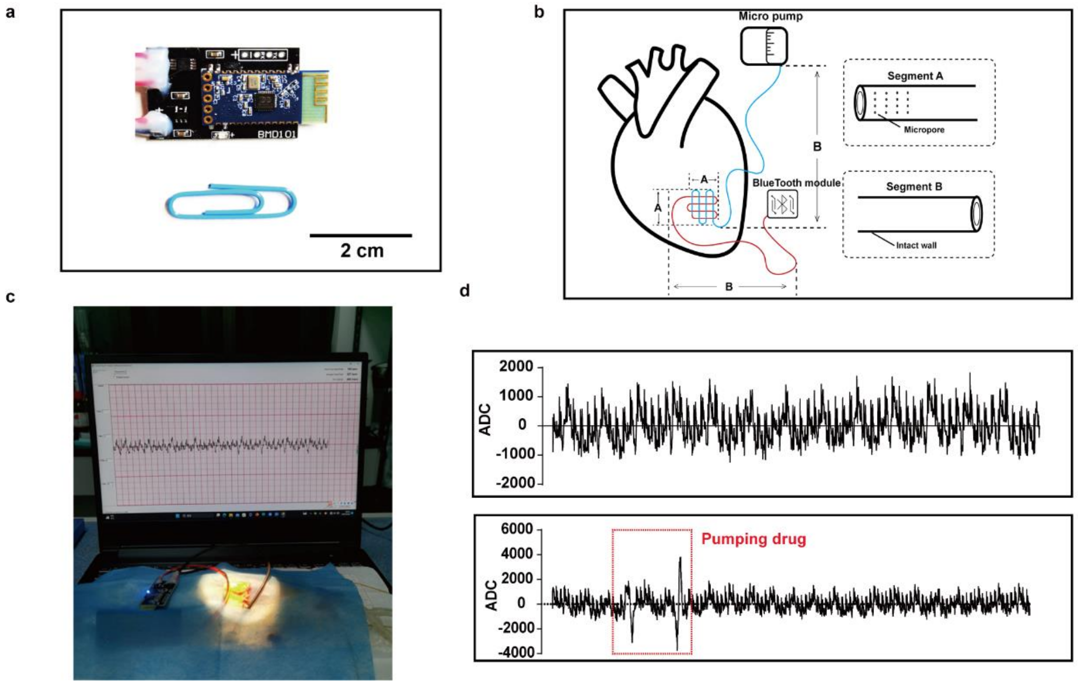

Figure S10. a The BMD101 Bluetooth module. **b** Schematic diagram of DTMS perfusion and sensing functions. **c** ECG signal measured by BMD101 chip while rat heart beating. **d.** ECG signal measured by BMD101 chip while rat heart beating. The red box represents the process of injecting drugs. In a very short time, the internal electrical signal was slightly disturbed, and then returned to normal.

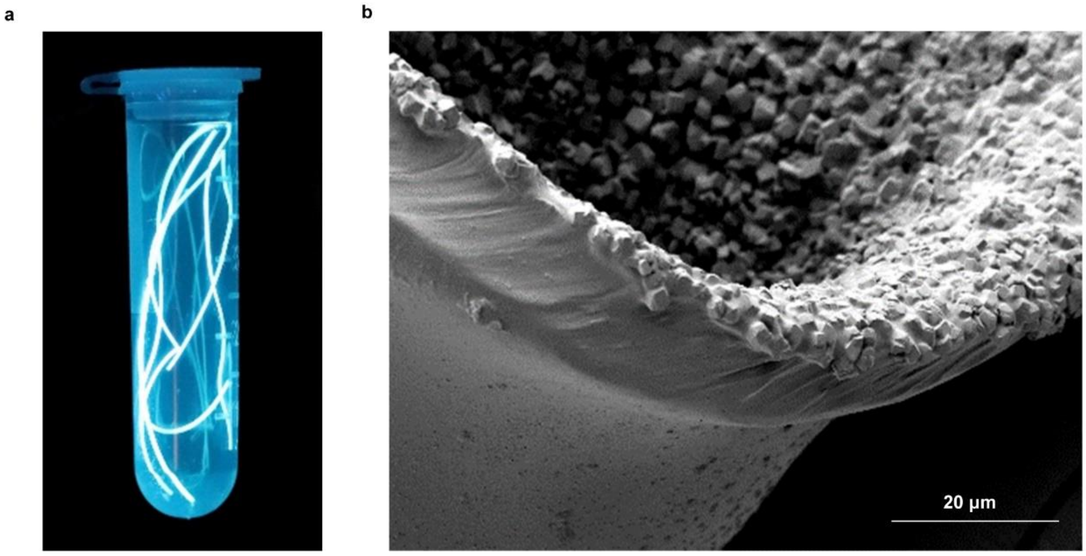

Figure S11. Electroluminescent DTMS characterization. a The DTMS contain 10% ZnS:Cu emits bright fluorescence under UV irradiation at 405nm wavelength. **b** Scanning electron microscope images of the cross-section of ZnS:Cu DTMS ; Bar=20μm.

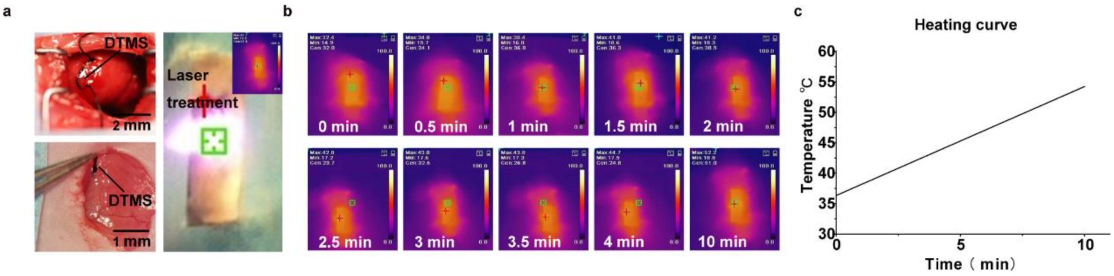

Figure S12. a NIR photothermal experiment of DTMS and PRIS in vitro. **b, c.** Heating curve of DTMS in vitro. The DTMS under skin was irradiated with 3w 808nm near-infrared laser at a distance of 25cm for 10min.

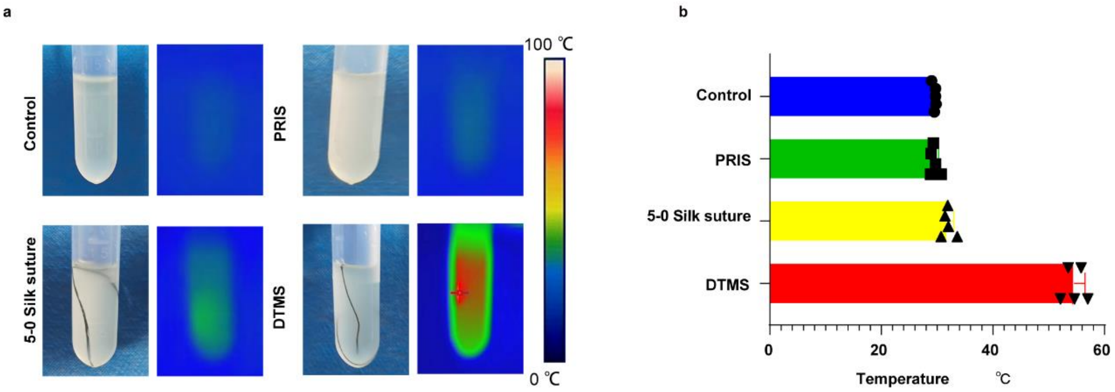

Figure S13. Photothermal effect of suture in Staphylococcus aureus solution: a Staphylococcus aureus expands one day in advance, 5-0 silk suture (Diameter: 200μm), DTMS suture (Outer diameter: 200μm, inner diameter: 80μm), PRIS (Outer diameter 200μm, inner diameter: 80μm), all suture lengths were 4cm; The thermal infrared signal was collected after 15 min of irradiation with

an 808nm NIR laser (Distance: 15cm, power: 3W). **b** The temperature of each group was counted. All values are presented as mean ± SD, n=5 independent replicates.

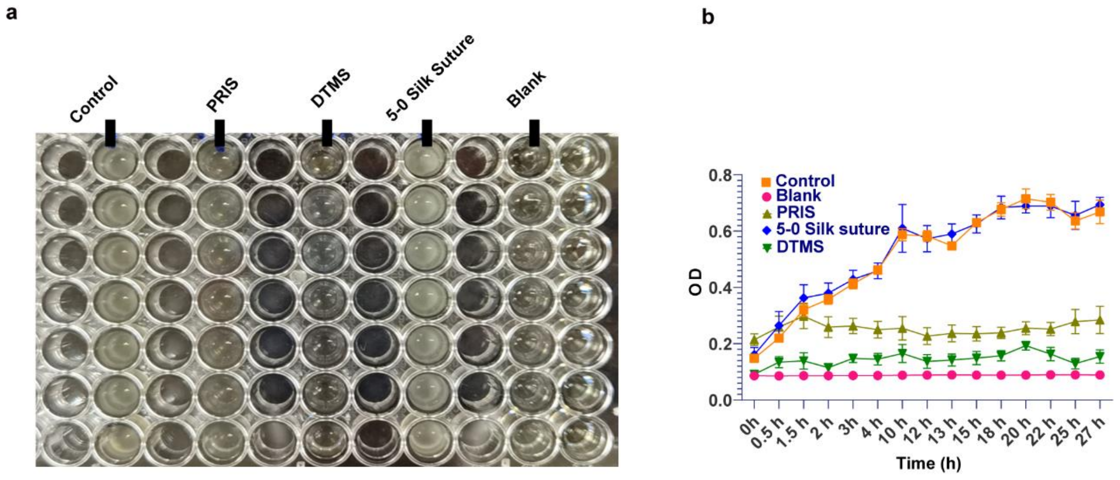

Figure S14. Photothermal bacteriostatic ability of sutures in Staphylococcus aureus solution. **a**After near-infrared laser irradiation, each group of Staphylococcus aureus solution was further cultured. **b** Over 24 hours of quantitative statistics of the absorbance at 600nm wavelength. All values are presented as mean ± SD, n=7 independent replicates.

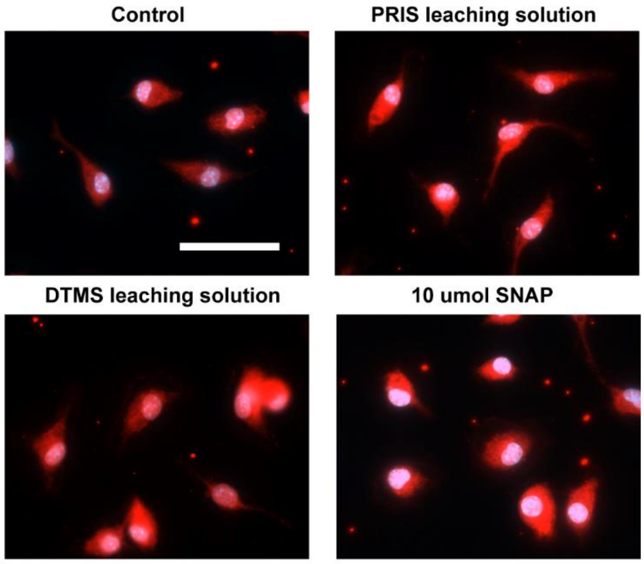

Figure S15. Rat BMSC cytoskeleton staining: Red: F-actin, blue: DAPI; BMSCs were treated with PVA and DTMS extracts and 10 μM snap for 48h. Bar=30μm.

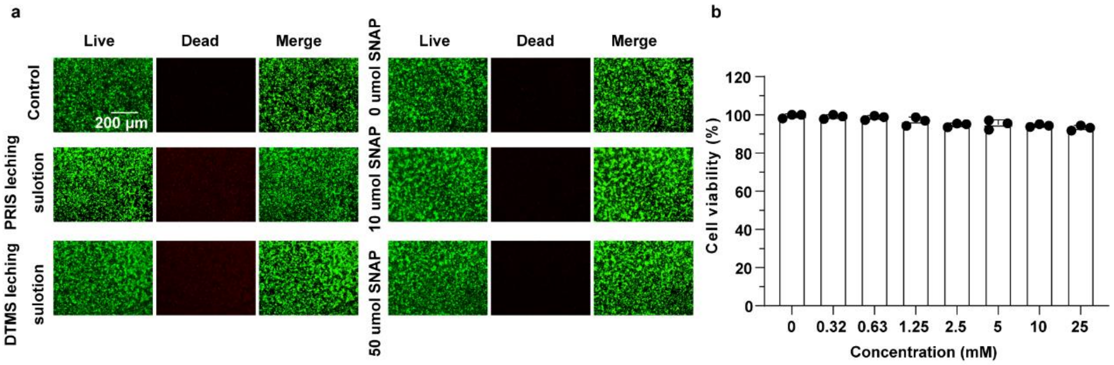

Figure S16. **a** HUVEC live and dead cell staining; All groups were treated for 48h and stained with Calcein and PI. **b** MTT colorimetry of the viability of HUVEC cells, all group was treated with gradient concentration of SNAP for 48h. All values are presented as mean ± SD, n=3 cell independent replicates.

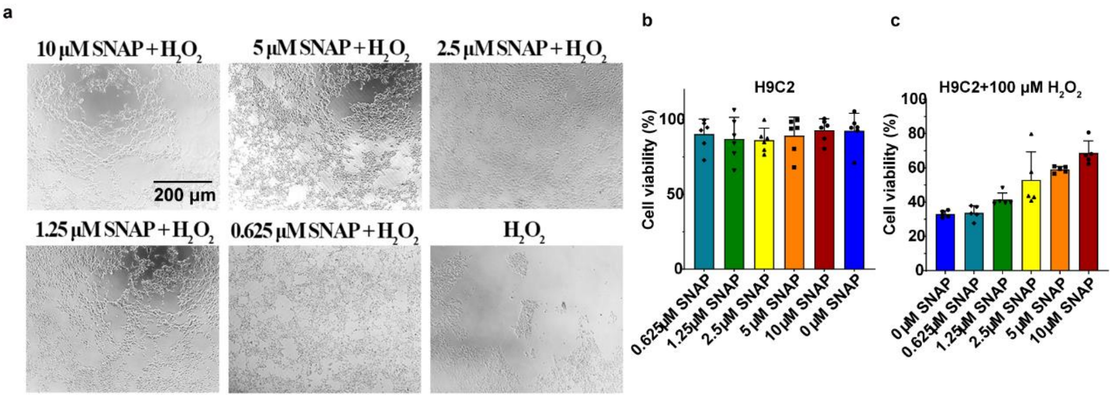

Figure S17: SNAP treated H9C2 cells with 100μM H_2O_2 for 24h. a SNAP and H_2O_2 co-incubate with H9C2. **b** MTT colorimetry of cytotoxicity of SNAP. All values are presented as mean ± SD, n=6 cell independent replicates. **c** MTT colorimetry of cytotoxicity SNAP + H_2O_2. All values are presented as mean ± SD, n=5 cell independent replicates.

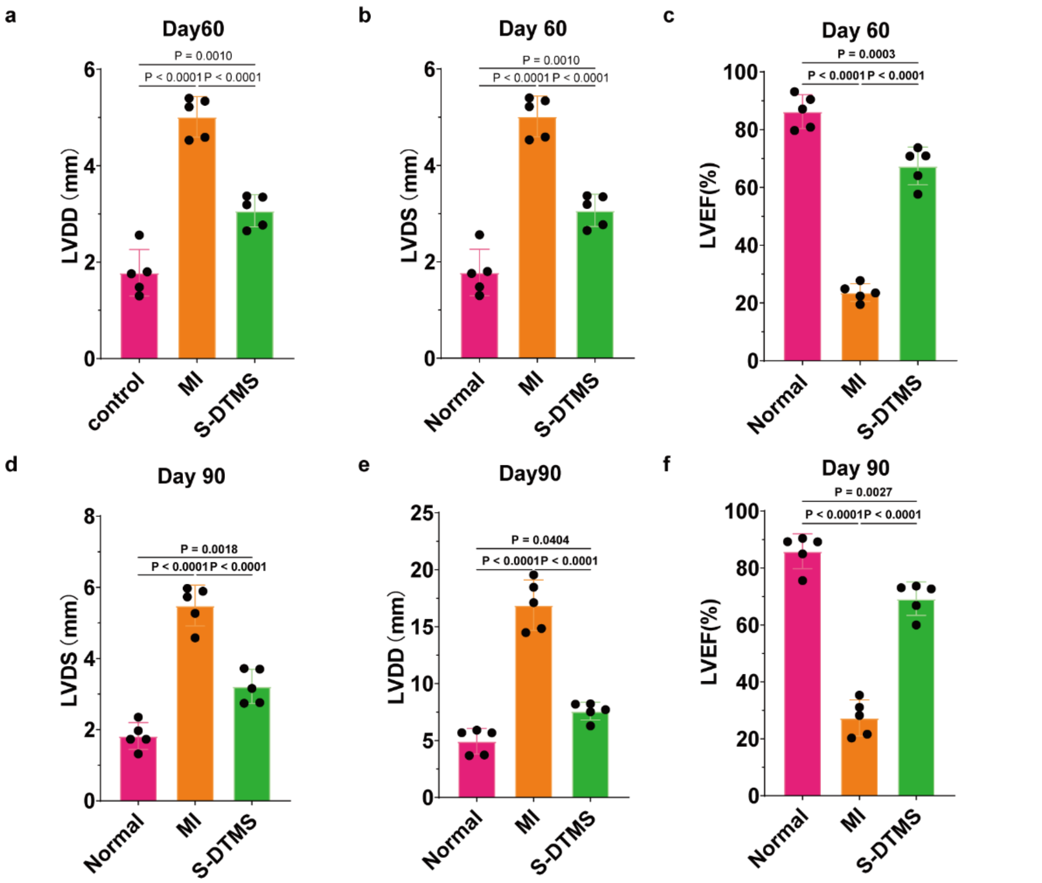

Figure S18. Cardiac function of rat. Quantitative analysis of LVDD, LVDS and LVEF evaluated by echocardiography on days 60**(a-c)** and 90**(d-f).** One-way ANOVA with multiple comparison tests. All values are presented as mean ± SD, n=5 biologically independent replicates.

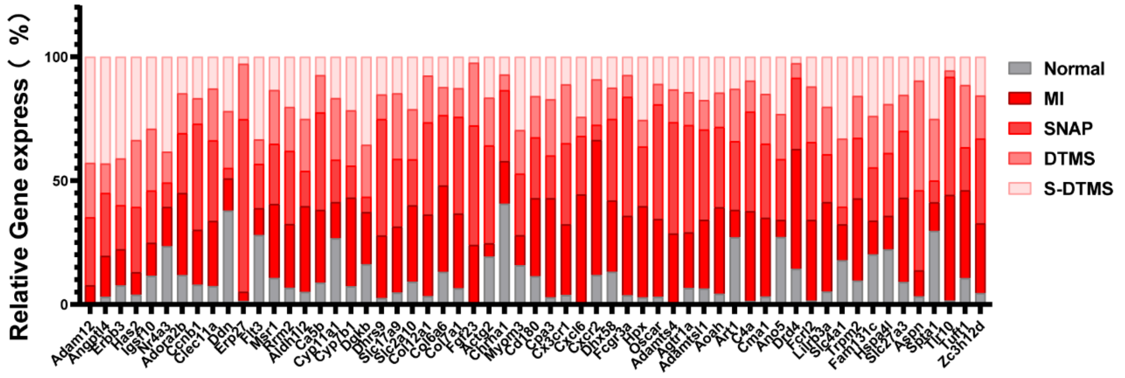

Figure S19. Quantitative analysis of myocardial cell related gene expression in infarcted area of rats.

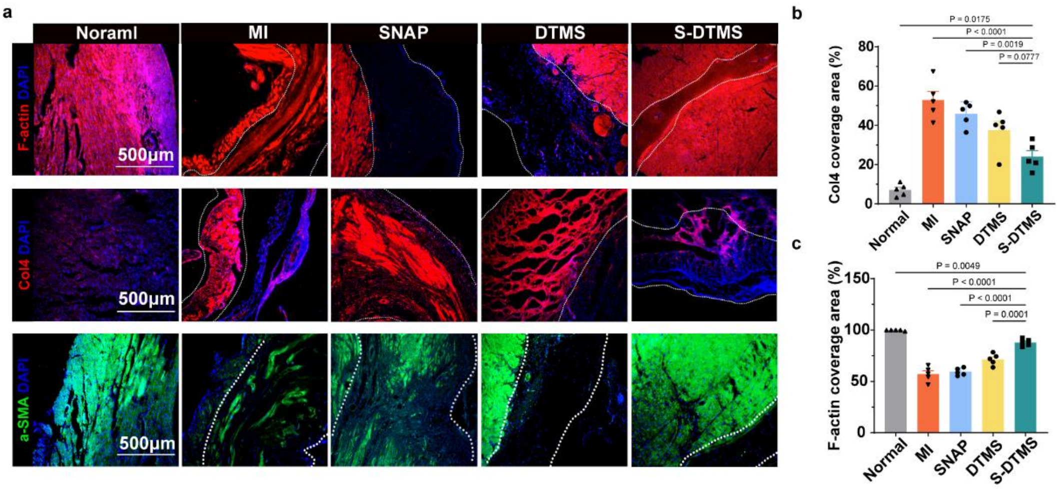

Figure S20. Immunofluorescence staining of myocardial tissue after 1 month treatment: a From top to bottom, F-actin (Red); Col4 (Red); α- SMA (Green), DAPI (Blue). **b, c** Statistics of F-actin and Col4 positive coverage area based on immunostaining images. One-way ANOVA with multiple comparison tests. All values are presented as mean ± SD, n=5 biologically independent replicates.

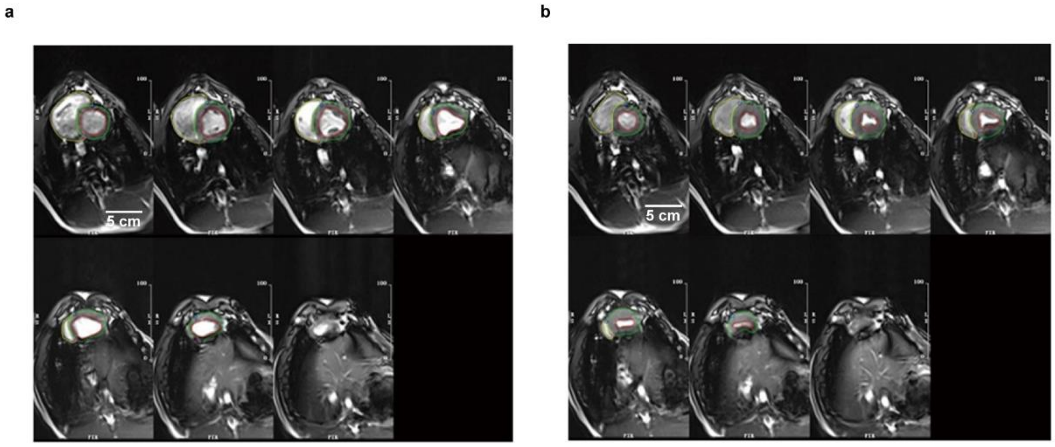

Figure S21： Normal group cMRI based on tissue feature tracking of cine sequence (a: diastole, b: systole)

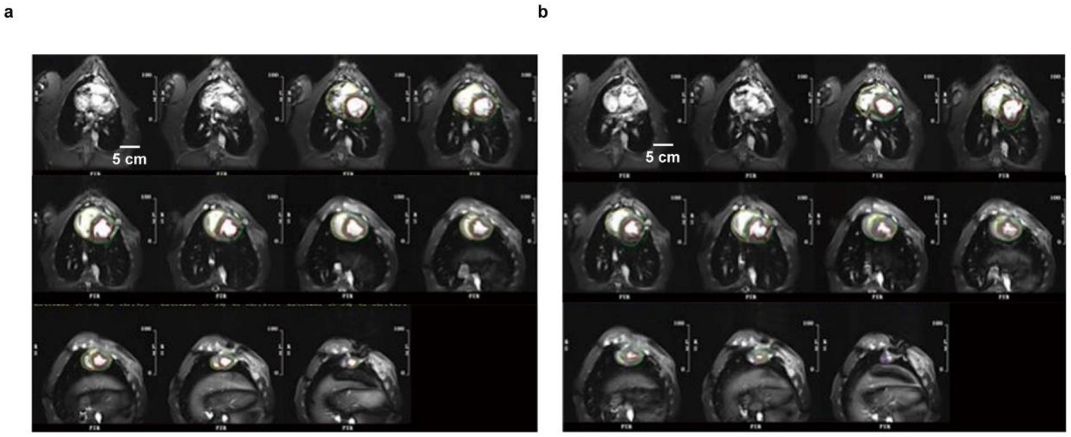

Figure S22: CMRI of MI group based on tissue feature tracking of cine sequence (a: diastole, b: systole)

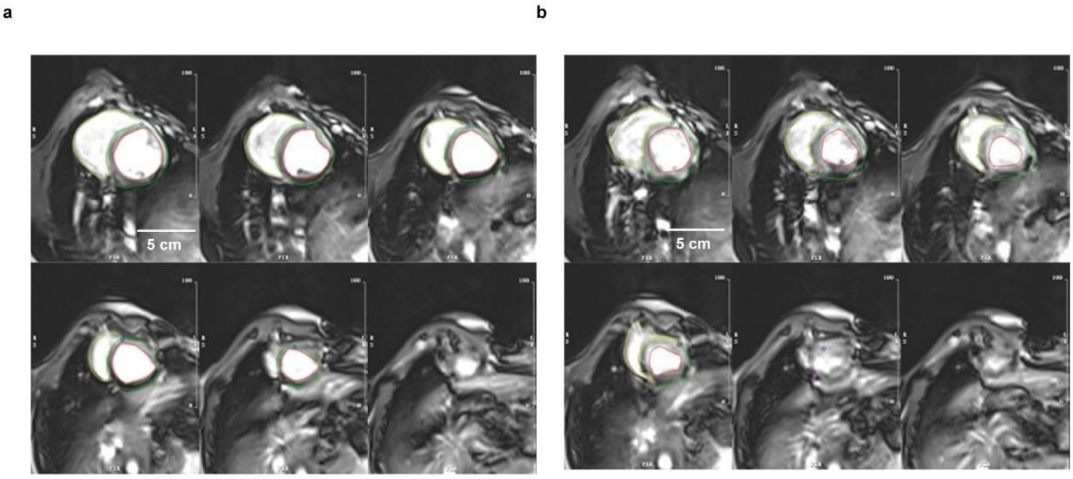

Figure S23: CMRI of SNAP group based on the tracking of tissue characteristics of film sequences (a: diastole, b: systole).

References:

[1] YeontaekLee *et al.*, A multifunctional electronic suture for continuous strain monitoring and on-demand drug release. *NANOSCALE* **13**.

[2] Ghosh, D. *et al.*, Light-Activated Tissue-Integrating Sutures as Surgical Nanodevices. *ADV HEALTHC MATER* **8** 1900084 (2019).

[3] Kalidasan, V. *et al.*, Wirelessly operated bioelectronic sutures for the monitoring of deep surgical wounds. *NAT BIOMED ENG* **5** 1217 (2021).

[4] Liu, M. *et al.*, Biomimicking Antibacterial Opto‐Electro Sensing Sutures Made of Regenerated

Silk Proteins. *ADV MATER* (2020).

5 Ma, Z. *et al.*, Bioinspired tough gel sheath for robust and versatile surface functionalization. *SCI ADV* **7** (2021).

6 Guan, Q. *et al.*, Bio-Inspired Lotus-Fiber-like Spiral Hydrogel Bacterial Cellulose Fibers. *NANO LETT* **21** 952 (2021).
