# Supplementary material for: Two way workable microchanneled hydrogel suture to diagnose, treat and monitor the infarcted heart
Source: Nat Commun. 2024 Jan 29;15:864. doi: 10.1038/s41467-024-45144-y (PMC10824767; doi:10.1038/s41467-024-45144-y)
Supplement: Supplementary file 3 — Description of Additional Supplementary Files [file 41467_2024_45144_MOESM3_ESM.pdf]

Description of Additional Supplementary Files

Supplementary Movies：

Supplementary Movie 1：DTMS manufacture. The aluminum wire with a diameter of 100 μm passes through a container filled with PVA-ppy solution, passed through motor rollers, dehydrated and collected on a hub.

Supplementary Movie 2: DTMS and PRIS's perfusion. DTMS (OD=200 μm, ID=100 μm) attached with a 34 G syringe and perfused the FITC solution, and the green, fluorescent solution passes through the DTMS.

Supplementary Movie 3: Force-induced luminescence of microchannel hydrogel. Microchannel hydrogel doped with 10% Cu:ZnS emits green light under cyclic stretching with frequency of 0.5, 1, 5, 10Hz.

Supplementary Movie 4: DTMS for rats' ECG signals transduction. DTMS was used for perception-treatment-monitoring after myocardial infarction in SD rats and demonstrated ECG on wireless devices.

Supplementary Movie 5: DTMS for diagnosis-treatment-feedback monitoring of MI in mini pigs.
