# Supplementary material for: Two way workable microchanneled hydrogel suture to diagnose, treat and monitor the infarcted heart
Source: Nat Commun. 2024 Jan 29;15:864. doi: 10.1038/s41467-024-45144-y (PMC10824767; doi:10.1038/s41467-024-45144-y)
Supplement: Supplementary file 9 — Reporting Summary [file 41467_2024_45144_MOESM9_ESM.pdf]

nature portfolio

Corresponding author(s): Wen Zeng Malcolm Xing

Last updated by author(s): Jan 10, 2024

Reporting Summary

Nature Portfolio wishes to improve the reproducibility of the work that we publish. This form provides structure for consistency and transparency in reporting. For further information on Nature Portfolio policies, see our Editorial Policies and the Editorial Policy Checklist.

Statistics

For all statistical analyses, confirm that the following items are present in the figure legend, table legend, main text, or Methods section.

n/a	Confirmed	
☐	☒	The exact sample size (*n*) for each experimental group/condition, given as a discrete number and unit of measurement
☐	☒	A statement on whether measurements were taken from distinct samples or whether the same sample was measured repeatedly
☐	☒	The statistical test(s) used AND whether they are one- or two-sided<br>*Only common tests should be described solely by name; describe more complex techniques in the Methods section.*
☐	☒	A description of all covariates tested
☐	☒	A description of any assumptions or corrections, such as tests of normality and adjustment for multiple comparisons
☐	☒	A full description of the statistical parameters including central tendency (e.g. means) or other basic estimates (e.g. regression coefficient) AND variation (e.g. standard deviation) or associated estimates of uncertainty (e.g. confidence intervals)
☐	☒	For null hypothesis testing, the test statistic (e.g. *F*, *t*, *r*) with confidence intervals, effect sizes, degrees of freedom and *P* value noted<br>*Give P values as exact values whenever suitable.*
☒	☐	For Bayesian analysis, information on the choice of priors and Markov chain Monte Carlo settings
☒	☐	For hierarchical and complex designs, identification of the appropriate level for tests and full reporting of outcomes
☒	☐	Estimates of effect sizes (e.g. Cohen's *d*, Pearson's *r*), indicating how they were calculated

Our web collection on statistics for biologists contains articles on many of the points above.

Software and code

Policy information about availability of computer code

Data collection	Sequencing data was colected by Illumina NovaSeq 6000 (Berry Genomics Corporation, Beijing, China),cvi42 software (Circle Cardiovascular Imaging Inc., Calgary, Alberta, Canada),ThingGear software, RM6240E-V2.5, Trans-PET BioCaliburn 700 system
Data analysis	GraphPad Prism9, 3DOSEM

For manuscripts utilizing custom algorithms or software that are central to the research but not yet described in published literature, software must be made available to editors and reviewers. We strongly encourage code deposition in a community repository (e.g. GitHub). See the Nature Portfolio guidelines for submitting code & software for further information.

Data

Policy information about availability of data

All manuscripts must include a data availability statement. This statement should provide the following information, where applicable:

- Accession codes, unique identifiers, or web links for publicly available datasets
- A description of any restrictions on data availability
- For clinical datasets or third party data, please ensure that the statement adheres to our policy

The RNA seq data from the present study are deposited in the National Center for Biofechnology information's Gene Expression Omnibus (accession number: GSE252825 (https://www.ncbi.nlm.nih.gov/geo/query/acc.cgi?acc=GSE252825)) . All other study data are included in the article and/or Supplernentary information. Source data are provided with this paper.

Research involving human participants, their data, or biological material

Policy information about studies with human participants or human data. See also policy information about sex, gender (identity/presentation), and sexual orientation and race, ethnicity and racism.

Reporting on sex and gender	N/A
Reporting on race, ethnicity, or other socially relevant groupings	N/A
Population characteristics	N/A
Recruitment	N/A
Ethics oversight	N/A

Note that full information on the approval of the study protocol must also be provided in the manuscript.

Field-specific reporting

Please select the one below that is the best fit for your research. If you are not sure, read the appropriate sections before making your selection.

☒ Life sciences ☐ Behavioural & social sciences ☐ Ecological, evolutionary & environmental sciences

For a reference copy of the document with all sections, see nature.com/documents/nr-reporting-summary-flat.pdf

Life sciences study design

All studies must disclose on these points even when the disclosure is negative.

Sample size	No statistical methods were used to predetermine the sample size. Sample sizes were chosen based on standard practice.
Data exclusions	No data were excluded.
Replication	All the reported experiments were reproducible. Data reproducibility was confirmed by independent experiments. All figure legends included repeat times.
Randomization	All animal- and cell-based samples in each of the group were included and no method of randomization was applied. As the results are qualitative, the randomization was not relevant in this study.
Blinding	Blinding is not relevant to our study

Reporting for specific materials, systems and methods

We require information from authors about some types of materials, experimental systems and methods used in many studies. Here, indicate whether each material, system or method listed is relevant to your study. If you are not sure if a list item applies to your research, read the appropriate section before selecting a response.

Materials & experimental systems

n/a	Involved in the study	
☐	☒	Antibodies
☐	☒	Eukaryotic cell lines
☒	☐	Palaeontology and archaeology
☐	☒	Animals and other organisms
☒	☐	Clinical data
☒	☐	Dual use research of concern
☒	☐	Plants

Methods

n/a	Involved in the study	
☒	☐	ChIP-seq
☒	☐	Flow cytometry
☒	☐	MRI-based neuroimaging

Antibodies

Antibodies used	Anti-CD31 Rabbit antibody [EPR17260-263] (Abcam, ab222783) ,1 500<br>Anti-a-SMA/ACTA2 mouse Antibody (Boster, Clone#1A4,BM0002),1 300<br>Anti-CD86 Rabbit antibody [EPR21962] (Abcam, ab239075),1 300

Anti-CD163 Rabbit antibody[EPR19518] (Abcam ab182422),1 300
Anti-Collagen I Rabbit antibody[EPR7785]? (Abcam,ab138492),1?500
Anti-Collagen III mouse antibody[FH-7A](Abcam, ab184993 ,1 500
Goat anti-Mouse IgG (H+L) Cross-Adsorbed Secondary Antibody, Alexa Fluor™ 488 Invitrogen A-11001 ,1 800
Goat anti-Rabbit IgG (H+L) Cross-Adsorbed Secondary Antibody, Alexa Fluor™ 568 Invitrogen A-11004 ,1 800

Validation

All antibodies are commercially available. Specific validation information and usage information can be found on their respective websites:
Anti-CD31 Rabbit antibody [EPR17260-263] (Abcam, ab222783) ,https://www.abcam.cn/products/primary-antibodies/cd31-antibody-epr17260-263-ab222783.html
Anti-a-SMA/ACTA2 mouse Antibody (Boster, https://www.boster.com.cn/index/products/productsDetail?goods_sn=BM0002,BM0002),
Anti-CD86 Rabbit antibody [EPR21962] (Abcam, ab239075),https://www.abcam.cn/products/primary-antibodies/cd86-antibody-epr21962-ab239075.html
Anti-CD163 Rabbit antibody[EPR19518] (Abcam ab182422),https://www.abcam.cn/products/primary-antibodies/cd163-antibody-epr19518-ab182422.html
Anti-Collagen I Rabbit antibody[EPR7785]? (Abcam,ab138492),https://www.abcam.cn/products/primary-antibodies/collagen-i-antibody-epr7785-ab138492.html
Anti-Collagen III Rabbit antibody[EPR17673](Abcam, ab184993), https://www.abcam.cn/products/primary-antibodies/collagen-iii-antibody-epr17673-ab184993.html
Goat anti-Mouse IgG (H+L) Cross-Adsorbed Secondary Antibody, Alexa Fluor™ 488 Invitrogen A-11001 ,https://www.thermofisher.cn/cn/zh/antibody/product/Goat-anti-Mouse-IgG-H-L-Cross-Adsorbed-Secondary-Antibody-Polyclonal/A-11001
Goat anti-Rabbit IgG (H+L) Cross-Adsorbed Secondary Antibody, Alexa Fluor™ 568 Invitrogen A-11011 ,https://www.thermofisher.cn/cn/zh/antibody/product/Goat-anti-Rabbit-IgG-H-L-Cross-Adsorbed-Secondary-Antibody-Polyclonal/A-11011

Eukaryotic cell lines

Policy information about cell lines and Sex and Gender in Research

Cell line source(s)

All cell lines purchased from ATCC: H9c2(2-1) (ATCC CRL-1446), HUVEC (ATCC PCS-100-010)

Authentication

No authentication for cell lines was performed.

Mycoplasma contamination

All cell lines tested negative for mycoplasma contamination.

Commonly misidentified lines
(See ICLAC register)

None of these cell lines were used in this study.

Animals and other research organisms

Policy information about studies involving animals; ARRIVE guidelines recommended for reporting animal research, and Sex and Gender in Research

Laboratory animals

SD Rat male,7-8weeks ,230-250g , MiniPig (male,1year old),The experimental rat were bred in house in SPF condition. Control rat were littermates housed with age and sex matched for each distinct rat strain. Rats were housed in cages with five rats per cage and kept on in a regular 12h light/12h dark cycle (lights on at 7:00 am). The temperature was 24±2 degree Celsius and humidity was 40-70%.

Wild animals

No wild animals were used in the study.

Reporting on sex

All the laboratory animals are male.

Field-collected samples

No field-collected samples were employed in this study.

Ethics oversight

Animals were purchased from the experimental animal center of the Third Military Medical University (license No. scxk (Yu) 2017-0002), all animal experiments were approved by the animal ethics committee of the Third Military Medical University (approval No.SYXK(Yu) 20170002 approval time: 2020.4.20). All experimental operations were performed in accordance with the guidelines for humane treatment of experimental animals.

Note that full information on the approval of the study protocol must also be provided in the manuscript.

Plants

Seed stocks	N/A
Novel plant genotypes	N/A
Authentication	N/A
